# Move to health-a holistic approach to the management of chronic low back pain: an intervention and implementation protocol developed for a pragmatic clinical trial

**DOI:** 10.1186/s12967-021-03013-y

**Published:** 2021-08-18

**Authors:** Daniel I. Rhon, Julie M. Fritz, Tina A. Greenlee, Katie E. Dry, Rachel J. Mayhew, Mary C. Laugesen, Edita Dragusin, Deydre S. Teyhen

**Affiliations:** 1 Department of Rehabilitation Medicine, Brooke Army Medical Center, JBSA Fort Sam Houston, 3551 Roger Brooke Drive, San Antonio, TX 78234 USA; 2 Department of Rehabilitation Medicine, Uniformed Services University of Health Sciences, Bethesda, MD USA; 3 University of Utah, Salt Lake City, UT USA; 4 Walter Reed Army Institute of Research, Silver Spring, MD USA

**Keywords:** Holistic health, Complementary and integrated health, Chronic pain, Low back pain, Health coach, Motivational interviewing, SMART goals, Behavioral health, Multidisciplinary care

## Abstract

**Background:**

The prevalence of chronic pain conditions is growing. Low back pain was the primary cause of disability worldwide out of 156 conditions assessed between 1990 and 2016, according to the Global Burden of Disease Study. Conventional medical approaches have failed to identify effective and long-lasting approaches for the management of chronic pain, and often fail to consider the multiple domains that influence overall health and can contribute to the pain experience. Leading international organizations that focus on pain research have stated the importance of considering these other domains within holistic and multidisciplinary frameworks for treating pain. While the research behind the theoretical link between these domains and chronic pain outcomes has expanded greatly over the last decade, there have been few practical and feasible methods to implement this type of care in normal clinical practice.

**Methods:**

The purpose of this manuscript is to describe an implementation protocol that is being used to deliver a complex holistic health intervention at multiple sites within a large government health system, as part of a larger multisite trial for patients with chronic low back pain. The Move to Health program developed by the US Army Medical Command was tailored for specific application to patients with low back pain and begins by providing an empirical link between eight different health domains (that include physical, emotional, social, and psychological constructs) and chronic low back pain. Through a six-step process, a health coach leverages motivational interviewing and information from a personal health inventory to guide the patient through a series of conversations about behavioral lifestyle choices. The patient chooses which domains they want to prioritize, and the health coach helps implement the plan with the use of SMART (Specific, Measurable, Attainable, Realistic, Time-bound) goals and a series of resources for every domain, triaged from self-management to specialist referral.

**Discussion:**

Complex interventions described in clinical trials are often challenging to implement because they lack sufficient details. Implementation protocols can improve the ability to properly deliver trial interventions into regular clinical practice with increased fidelity.

**Trial registration:**

Implementation of this intervention protocol was developed for a clinical trial that was registered a priori (clinicaltrials.gov #NCT04172038).

**Supplementary Information:**

The online version contains supplementary material available at 10.1186/s12967-021-03013-y.

## Introduction

### Chronic low back pain in the military health system

Chronic pain is a ubiquitous and growing concern in the Military Health System (MHS) as it is in civilian health systems. Incident rates of chronic pain for active-duty military members have increased more than threefold in recent years [1]. The most common chronic pain condition seen in the MHS is low back pain [2, 3], accounting for about 70% of medical encounters for chronic pain in active duty military members [1]. Low back pain (LBP) has been the most common reason for a medical encounter in the MHS every year since 2011, accounting for over 1 million encounters in 2015 [4]. In the MHS, LBP is also the most common diagnosis for which opioids are prescribed [5] and the leading cause of medical discharge across all military services [6]. Improving care for LBP is a priority consideration for pain management in the MHS [1].

Chronic pain, particularly LBP, is often accompanied by a large burden of comorbid conditions, high levels of psychological distress, and unhealthy lifestyle habits; all of which increase risks for persistent disability and delayed return to full duty for military personnel [7–11]. Recognition of the multi-dimensional nature of chronic pain has increased emphasis on a biopsychosocial perspective and a more holistic treatment approach [12]. A biopsychosocial approach to chronic LBP is reflected in current practice guidelines in civilian [13, 14] as well as the Military and Veterans’ Health Administration (VHA) systems [15]. These guidelines are consistent in recommending first-line care focused on nonpharmacologic treatment to promote self-management while advising against analgesic medication, particularly opioids, and spinal imaging as first-line options.

Despite current practice guidelines, clinical care remains stubbornly grounded in a biomedical paradigm focused on identifying and fixing a presumptive pathoanatomical cause [16]. Challenges in transitioning from a biomedical to biopsychosocial paradigm are evident in both civilian health systems and the MHS; with persistently high rates of low-value care including prescription opioids, imaging and interventional pain procedures [17]. A biopsychosocial paradigm would place greater emphasis on connecting patients with population health resources for self-management with focus on the importance of maintaining physical activity and addressing the role of psychological and social factors as barriers or facilitators [17]. Implementing interventions that address this broad spectrum of factors can be challenging, and requires clear guidance and transparency with treatment descriptions in order to maximize replication.

### Move to health in the military health system

In 2010, the Army Pain Management Task Force recommended a person-centered approach to empower individuals to participate in their care, with greater emphasis on the biopsychosocial impact of pain [18]. These recommendations coincided with efforts to transform Army Medicine from a traditional health care system to a System for Health that maintains, restores and improves health through physical, emotional, social and spiritual fitness [19]. The Office of the Army Surgeon General developed several strategies to facilitate the transition to a System for Health including “Move to Health” [20], which is built on the “Whole Health” program in the VHA [21]. Whole Health is focused on transforming health care delivery to embrace person-centered, holistic care emphasizing the power of self-management to strengthen innate healing capacities using both complementary and integrative health (CIH) approaches and population health resources [21]. Complementary and alternative care refers to interventions that are not considered conventional or usual Western practice. Integrated health refers to the collation of conventional and complementary approaches together in a coordinated manner [22]. Population health refers to the determinants of health outcomes for a community such as the physical and social environment and available resources [23].

**Fig. 1:**
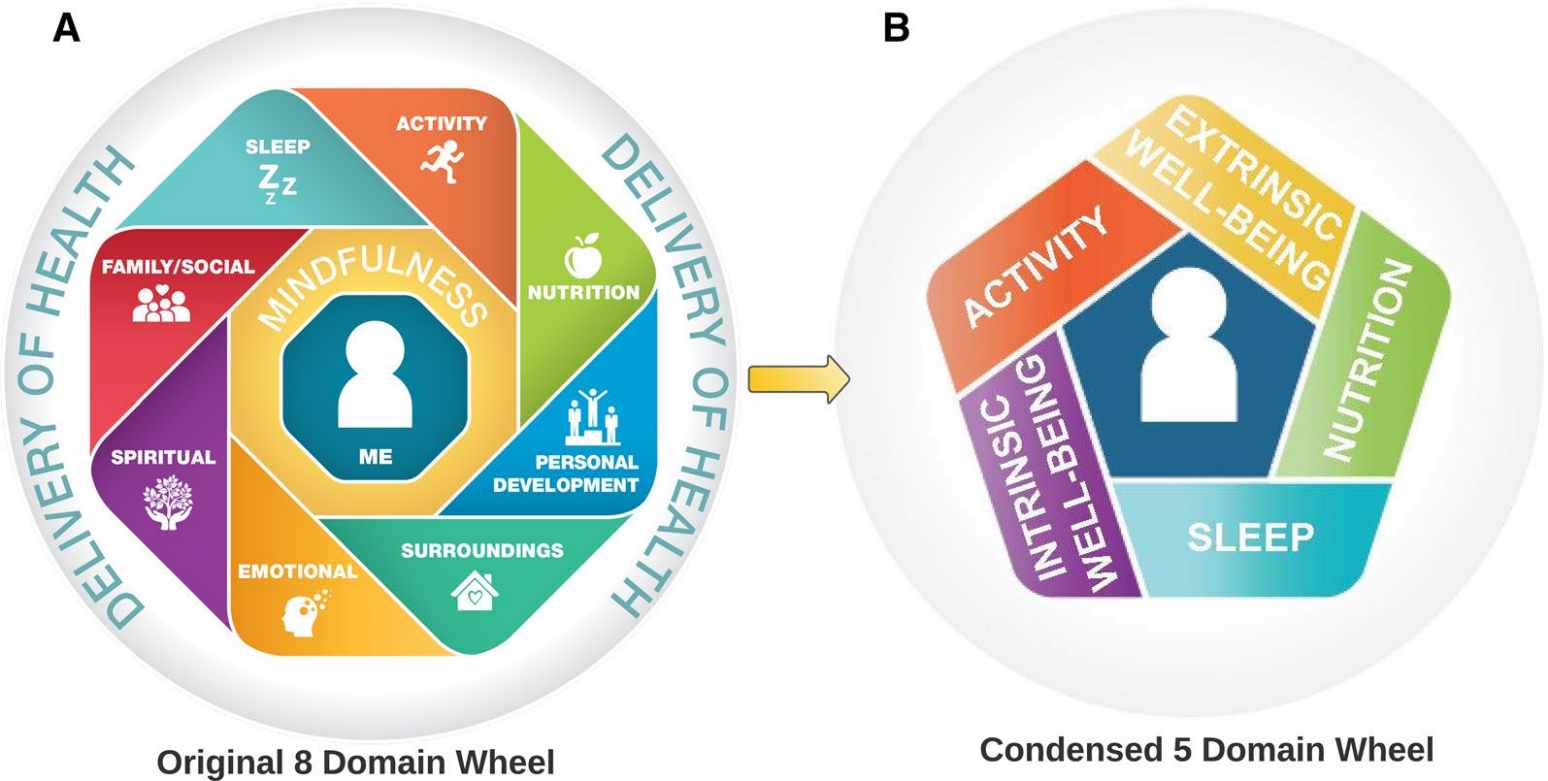
Move to Health Wheel. **A** Original move to health wheel with all 8 domains. **B** 5-domain wheel where emotional, spiritual, personal development, family/social relationships, and surroundings were consolidated into “Intrinsic” and “Extrinsic” well-being

The Move to Health (M2H) program represents a comprehensive approach to shift from a disease-focused, biomedical model towards a person-centered strategy to optimize well-being through engaged patients who are empowered to improve their own health [20]. The concept is best visualized through the M2H wheel, where the components of the wheel represent the various health domains, and at the center is the individual person (Fig. 1a). A fundamental tool for maintaining person-centeredness within M2H, is a personal health inventory (PHI; Additional file 1: Appendix SA1). The PHI facilitates identification of personal health goals with consideration of the M2H wheel self-care domains including sleep, physical activity, nutrition, intrinsic well-being (includes emotional, mental and spiritual health, personal development and struggles with addictive behaviors, including tobacco, alcohol and other substances) and extrinsic well-being (family, social relationships and an individual’s home and work environments). Achieving personal health goals depends on two key components. First, a health delivery system characterized by collaboration between traditional medical, CIH and population health resources that support the person and provides evidence-based care focused on health and self-management instead of disease management [24]. And second, establishes a healthy environment including access to self-management resources and support for healthy lifestyles. With the help of these tools and resources, the M2H program leverages evidence-based practices to help enable behavior change, to include motivational interviewing, positive psychology, and appreciative inquiry [25].

### Move to health and chronic low back pain

The M2H program, with its focus on holistic, person-centered care, is highly aligned with the need to transition chronic LBP management from a biomedical to a biopsychosocial paradigm. Adapting M2H to meet the needs of individuals with chronic LBP could be an important tool for closing the gap between clinical care and evidence-based recommendations emphasizing nonpharmacologic options and promotion of self-management as first-line treatments. In the VHA, the Whole Health program has been piloted as a strategy to improve pain management in light of the national opioid epidemic with encouraging results [24]. Similarly, operationalizing M2H as a strategy to improve chronic LBP management is well-aligned with MHS priorities and could have a substantial positive impact.

We are conducting a randomized clinical trial as part of the NIH-DoD-VA Pain Collaboratory [26]. The Collaboratory is an unprecedented effort of federal agencies to build capacity for large-scale pragmatic clinical research in the MHS and VHA focused on improving pain management through nonpharmacologic care. Our team’s clinical trial investigates the implementation of pragmatic, first-line treatment strategies for chronic LBP in the MHS including M2H as an intervention arm [27]. Developing the study protocol required operationalizing M2H for chronic LBP around the key components outlined above (using a PHI and goal setting, developing management algorithms for collaborative care between traditional and CIH and population health resources, and facilitating self-management). The purpose of this paper is to describe the development of the M2H program as a strategy to promote the biopsychosocial management of individuals with chronic LBP.

### Move to health domains and chronic low back pain

Tailoring M2H for chronic LBP management necessitates helping patients make connections between M2H health domains and their LBP, then facilitating the capacity for self-management. Many individuals with pain may not recognize that the domains in the M2H model (Fig. 1) are relevant to the pain experience [10, 28–35]. Helping persons with LBP understand the connections between holistic aspects of health and pain is foundational to the application of M2H as a strategy to manage chronic LBP.

The original M2H wheel comprised eight health domains for patients [20]. We condensed these into five larger domains to facilitate implementation (five intervention approaches; Fig. 1b). We maintained the domains from the Performance Triad [36], a component of the M2H program focused on the important roles of sleep, nutrition and physical activity. The other domains (emotional, spiritual, personal development, family and social relationships, and surroundings) were consolidated into the categories of intrinsic and extrinsic well-being. The wheel is a tool used by the health coach to broaden the conversation about these health domains. It is important to note that interactions across domains are common. Positive or negative change in one domain can influence the others and may indirectly influence pain-related symptoms. For example, regular physical activity is associated with greater total sleep time, sleep efficiency and overall sleep quality [37, 38]. Several nutritional interventions have been shown to improve sleep [39], and good sleep can improve cognition (which influences pain [34]) and emotional state [40, 41]. Family and social relationships can have goals that focus on either intrinsic and extrinsic well-being, or both. Additional details about the health domains as they relate to chronic LBP are outlined below.

#### Sleep

A negative association between disturbed sleep and chronic LBP is well established [42]. More than 50% of those with chronic LBP report sleep disturbance [43–46] Sleep deprivation has been connected with heightened pain sensitivity [47] and elevated inflammatory markers [48, 49]. Sleep disturbance increases the risk that acute LBP will become chronic, worsens the prognosis for chronic LBP [50, 51], and is common among service members. A 2015 survey of over 16,000 service members found 30% were moderately or severely bothered by lack of energy due to poor sleep, 56% got less sleep than they needed, and 9% reported using sleep medication daily or almost every day [52].

#### Physical activity

Greater physical activity is associated with reduced risk for developing chronic LBP [53, 54], and physical activity is an effective treatment for individuals with LBP [55]. Current guidelines establish early physical activity as a core recommendation [13, 15, 56, 57]. Many persons have low levels of regular physical activity, and surprisingly this also includes service members [58, 59]. Persons with chronic LBP experience additional barriers to physical activity including pain and concerns about re-injury or worsening their condition [60, 61]. Facilitating physical activity for those with chronic LBP often requires addressing maladaptive beliefs as well as addressing motivation and other typical barriers to engaging in regular physical activity [62].

#### Nutrition

Deficient nutrient intake, obesity, and poor eating behaviors have been reported in patients with chronic pain [63] and dietary changes aimed at reducing body fat or adapting a healthier diet can have a positive impact on chronic pain [64, 65]. Diet has been linked to central and peripheral pain pathways, [66, 67], and though the mechanisms linking chronic pain and diet are not clear, it increasingly appears that diet and weight management are part of a holistic strategy to manage chronic pain [68, 69]. Hydration levels can influence pain perception, highlighting the importance of proper fluid intake [70, 71]. Among service members, 13% of active duty personnel are obese, and only 13% report meeting all targets for national nutrition standards [52].

#### Intrinsic well-being

This domain includes aspects of an individual’s emotional and spiritual life and personal development, which impact overall well-being and the pain experience. There is a body of literature supporting relationships between personal psychological and personality characteristics and chronic pain. Catastrophic thinking or fear of pain can amplify the pain experience and promote chronification, while factors such as resilience and optimism are associated with improvement in pain and less disability [72–74]. Spirituality can contribute to a sense of value, resilience, meaning and purpose, and can play a role in pain-related beliefs and coping responses of patients with chronic pain [75]. Mindfulness, or intentional and nonjudgmental awareness of present experience, can reduce pain and enhance function for individuals with chronic LBP likely through enhanced sense of control over pain and reduced catastrophizing [76, 77]. Additional personal habits and maladaptive stress responses such as smoking can also increase risk for persistence of chronic LBP [78]. Much of this emotional state can also stem from past trauma or abuse.

#### Extrinsic well-being

Research in individuals with chronic LBP reveal that an individual’s environment, including work, family or other external influences, impact the pain experience. Factors such as marital quality, perceived social support and supportive work environments can hasten recovery from LBP and reduce disablement [79–83]. Additionally, the behaviors and attitudes of one’s spouse or partner towards healthy lifestyle choices such as physical activity, smoking and weight management are highly influential [84–86]. Service members span from a large diversity of settings and backgrounds, raising the possible connection between social determinants of health prior to military service and their influence on current extrinsic well-being.

### Operationalizing move to health for chronic low back pain

**Fig. 2:**
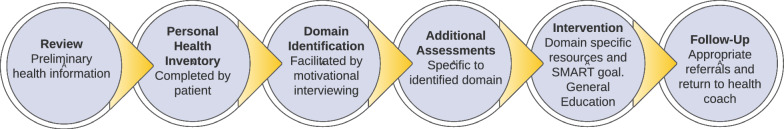
Process steps for the M2H intervention for patients with chronic low back pain

**Fig. 3:**
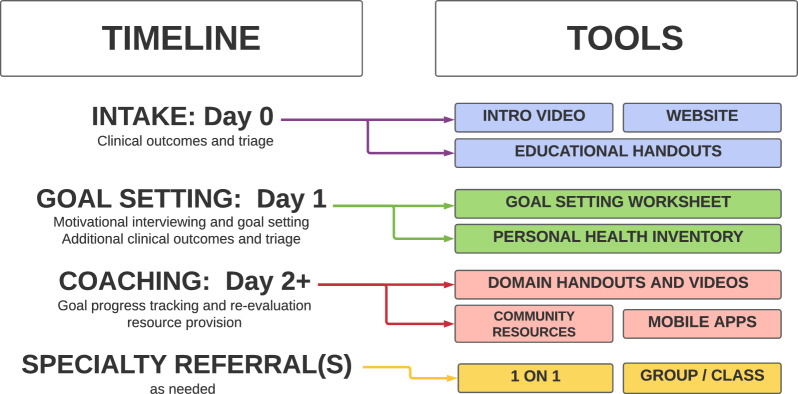
Integration of program in clinical practice and suggested timeline for events

Engaging a person with chronic LBP in the M2H program is led by a trained health coach. The goal of the coach is to assist in the identification of health domains that impact a person’s pain experience and empower them to make changes. A 6-step process is used to achieve this (Fig. 2). Integration of the approach in clinical practice and suggested timeline are outlined in Fig. 3. We provide a brief summary of each step below.

#### Step 1: review of health information

The first step in the M2H process is a review of the person’s health information and background. The purpose of this step is to help the coach identify health domains that may relate to the person’s pain experience. In the context of our PMC trial, we use various self-report measures and questionnaires to obtain this health information (Table 1). Other settings may have different measures for the constructs listed or have measures of additional constructs that may help the coach identify opportunities for improved health.

**Table 1:** Baseline factors pertinent to M2H domain identification and approach

Variable	Measures	Interpretation for move to health
Body mass index	Height and weight (kg/m^2^) [87]	Can suggest an opportunity to improve health through domains of nutrition and/or activity < 18.5 = underweight; 25–29.9 = overweight; ≥ 30 = obese
Tobacco use	History of tobacco use	Can suggest an opportunity to improve health through the domain of personal development in the intrinsic well-being category
Physical activity	Godin Leisure-Time Questionnaire [88]	Can suggest an opportunity to improve health through the domain of activity 24 + : Active; 14–23: moderately active; < 14: insufficiently active/sedentary
PROMIS health domains Sleep disturbance [89] Depression [90, 91] Anxiety [92] Pain interference [93]	PROMIS short forms or computer-adapted tests [92]	PROMIS domains are provided as T-scores. For the following domains scores from 55.0 to 59.0 suggest a mild concern, 60.0–69.0 suggest a moderate concern, and scores ≥ 70 suggest a severe concern Suggests an opportunity to improve health through the domain of sleep Both depression and anxiety may suggest opportunities to improve health in the domains of intrinsic or extrinsic well-being Examines impact of pain on mood, stress, sleep and activity, suggests opportunities to improve health in the intrinsic or extrinsic well-being categories, sleep or activity
Physical function	PROMIS physical function [92, 94]	Suggests opportunity to improve health through the domain of activity
Pain impact	PEG-3 [93] or Defense and Veterans Pain Rating Scale [95]	Pain impact combines measures of pain intensity and pain interference. suggests opportunities to improve health in the intrinsic or extrinsic well-being categories, sleep or activity Both the PEG-3 and DVPRS are scored from 0–10 with higher numbers indicating greater pain impact
Prognosis	Keele STarT Back Screening Tool [96]	Risk level provides prognosis of poor clinical outcomes and suggests opportunities to improve health in the domains of emotional (intrinsic) well-being and activity Total score ≤ 3 points = low risk; total score ≥ 4 points (≤ 3 points on Qs 5–9 = medium risk; 4 + points on Qs 5–9 = high risk)
Health-related quality of life	EuroQol-5D-5L [97, 98]	Suggests opportunity to improve health in the domain of emotional, spiritual, personal development, family/social, or surroundings in the intrinsic or extrinsic well-being categories Overall health is self-reported 0 (Worst) to 100 (Best); patients report dimension-specific difficulties with mobility, self-care, usual activities, pain/discomfort, and anxiety/depression from 1 (no problems) to 5 (unable to or extreme problems)

*PROMIS* patient-reported outcomes information system, *PEG-3 *pain average, enjoyment of life, and general activity, *EuroQol *european quality of life 5 dimension, 5 level scale

#### Step 2: personal health inventory

Following review of health history, the person with LBP views an introductory video linking M2H to LBP (https://vimeo.com/498500657) and then the PHI (SA1) is completed. The PHI asks the person with LBP to reflect on each domain of the M2H wheel (Fig. 1) and rate where they are currently; and where they would like to be for each domain on a 1–5 scale. The person is asked to list actions they may want to take to reach their goal within a domain. It may be helpful to have the person with LBP complete the PHI at home following an initial introduction to M2H. This may allow persons to reflect on the questions on the PHI and how the M2H domains may connect to their health.

#### Step 3: domain selection

After the PHI is complete, the health coach reviews the responses with the person. Considering the responses on the PHI and information gathered from the review of health information, the health coach and person with LBP collaborate to identify a priority domain for which the person’s motivation is strongest for making a lifestyle change. The health coach uses principles of motivational interviewing to engage and assist the person with LBP to identify a priority domain. Motivational interviewing (MI) is an evidence-based method to support persons who are uncertain or ambivalent about setting health goals or taking steps towards achieving them [99]. An MI approach uses empathy and nonjudgmental inquiry to build motivation for change for the person with LBP [100] and is consistent with the goal of making care person-centered and focused on self-management.

#### Step 4: additional assessments

Once a priority domain is selected, additional assessments may be used to help identify resources and/or referrals that may benefit the person in making a desired lifestyle change. Responses to additional assessments are consolidated with the health information from Step 1 into a Summary Report that includes scoring interpretation guides and is used for quick reference by the health coach (SA3). The Summary Report helps the health coach connect the person with LBP to traditional, CIH and population health-based resources (Table 2).

**Table 2:**
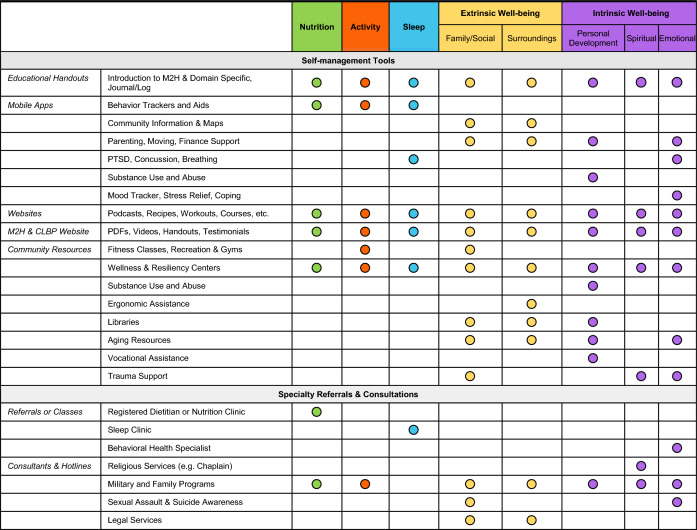
Resources available matched to each Move to Health Domain

Dots indicate primary usage of given resources in a military population with chronic low back pain

For the sleep domain, additional assessments are used to identify issues of insomnia, daytime sleepiness, sleep apnea or sleep disturbances related to psychological conditions including depression or post-traumatic stress disorder. The presence and severity of these sleep disorders may necessitate referral to sleep medicine specialists in the case of sleep apnea, or a behavioral health provider if disturbed sleep is attributable to psychological factors. Other conditions of disturbed sleep may be managed by the health coach. The additional Advanced Sleep Screen algorithm and questionnaires are provided in the Additional file 1: Appendix (SA2) and help guide the health coach with their decisions about when referrals to a sleep specialist may be necessary.

For the physical activity domain, additional assessments include the Fear Avoidance Questionnaire physical activity subscale (FABQ-PA [101]) and the Godin Leisure-Time Exercise Questionnaire (GLTEQ) [88, 102]. The FABQ-PA helps to identify fear and avoidance behavior interfering with physical activity. The GLTEQ helps to classify individuals into categories of active or insufficiently active. These questionnaires can assist with goal setting and M2H education.

In the nutrition domain, the Healthy-Eating Score-5 is used for additional assessment and assistance in setting goals around healthy eating habits [103]. All persons identifying the nutrition domain are offered consultation with a dietician to assist with identifying problematic nutrition habits and goals, particularly for individuals who may have comorbid health conditions that require special dietary considerations such as diabetes.

If the domain of intrinsic or extrinsic well-being is of interest to the patient, the health coach will help them identify the appropriate referral based on their area of concern. Referrals may include a spiritual advisor/counselor, behavioral health provider, smoking cessation resources or primary care provider if concerns for high levels of untreated anxiety or depression are identified.

#### Step 5: goal setting and intervention

After the health coach assists the patients with identification of a priority domain, the next step is setting an initial goal. Goal setting is an important component of motivating behavior change [104]. The health coach uses MI principles in guiding the patient to set a goal that is intrinsically meaningful and achievable, which further enhances motivation to change [105]. MI leverages open-ended questions that seek to identify patient-driven motivation to change behavior by enabling a discussion about the strengths and barriers to reaching those goals. This in turn helps establish confidence and promote self-efficacy around the ability to meet these goals and is a necessary step for developing a detailed action plan to prepare the person for success. The model used for the goal setting exercise is SMART: specific (exactly what will be achieved), Measurable (how to know if it has been achieved), Attainable (skills and abilities to achieve based on current circumstances), Realistic (considers available time and financial resources), and Time-bound (when the goal will be accomplished). Setting actionable goals has been found to help individuals make healthy behavior changes including smoking cessation, healthier eating, physical activity, etc. [106]. An example of a relevant SMART goal is found in Table 3, and the M2H worksheet for SMART goal setting is provided in the Additional file 1: Appendix (SA5).

**Table 3:** Example for Crafting a Relevant SMART Goal

General goal: incorporate more yoga into my routine	
S: specific	Online vinyasa yoga videos
M: Measurable	45 min; 2 times per week
A: Attainable	Currently doing 45-min video once per week
R: Realistic	Free video; wi-fi access; time available on a second day during the week
T: Time-bound	For the next 4 weeks

SMART Goal: incorporate a 45-min, online vinyasa yoga video into my routine 2 times per week for the next 4 weeks

Once a SMART goal is set, the health coach provides resources to supplement any referrals that may be appropriate to the chosen domain and SMART goal. The health coach first provides educational resources that are standardized for all M2H participants with chronic LBP (SA6). These resources outline the importance of the holistic approach to health used in the M2H program as well as instructions in a walking program for physical activity, sleep positioning for individuals with LBP and deep breathing exercises for relaxation and mindfulness. The health coach provides an overview of each educational handout and engages with the individual to answer questions about the topic of each handout.

Next, the health coach provides resources specific to the domain selected by the patient and their SMART goal (Table 2). These include educational materials from the M2H program modified for a more specific focus on chronic LBP (SA5-S8 and move2health.org). The types of resources include handouts (SA6), mobile apps (SA7), videos from the Performance Triad initiative (https://p3.amedd.army.mil/), and government or trusted medical organization websites (SA8). The health coach also maintains a list of local health system and community resources that can assist each person in working towards their goal. The interventions for each domain were developed within a stepped-care framework with the goal of having a range of options available, and the health coach strategically introduces them as needed so as to not overwhelm the patient (additional resources can be provided at follow-ups). For example, a person could identify sleep or nutrition as a primary goal, and the initial steps would revolve around self-management strategies. However, this person may eventually receive a consultation with a sleep specialist or have a 1:1 visit with a registered dietician if needed to meet their goal.

#### Step 6: follow-up

After establishing the initial treatment plan directed towards the person’s SMART goal a follow-up session is typically planned after 1–2 weeks. Purposes of follow-up are to provide an opportunity to ask questions about M2H, check on progress towards goals, provide an opportunity to identify new or additional domains and/or SMART goals, and assist with setbacks or difficulties encountered. At each follow-up a determination is made to continue, modify, or retire any current goal. The person with LBP should be working on no more than 2 goals at any one time. Generally, goals are retired once they are achieved, the person wants to change their focus, or a level of self-efficacy is achieved, and the person feels comfortable with continued pursuit of the goal independently. Weekly follow-ups are standardized (SA4) in order to guide the health coach and ensure specific topics are covered. Follow-up sessions are rooted in three concepts: M2H, MI and reviewing progress towards SMART goal achievement. Once the person with LBP is satisfied with the progress on all of their goals, and ready to transition to continued independent maintenance of their health, health coaching can be discontinued. The individual is free to return at any time in the future to reassess their goals and establish a new plan if desired.

### The role of mindfulness in move to health

Mindfulness is also considered a core component of the M2H program, and is labeled specifically in the center of the original M2H wheel alongside the individual person to show that mindfulness is used to guide their interaction with each domain [20], providing a strategy that the person can use to focus their attention on the implications of each domain presented to them. Mindfulness is another tool to supplement the MI approach to enabling behavior change. Mindfulness can become a foundation from which the person is more prepared to engage with the changes in behavior that are the ultimate goal of the M2H program. Acceptance and mindfulness programs are effective for chronic pain [77] and are thought to work through mechanisms of changing perceptions of pain control and pain catastrophizing [76]. Mindful breathing has been shown to reduce pain perception in a variety of different populations [107]. The M2H program does not use a formal mindfulness protocol, but rather the health coaches were trained to introduce the concept of mindfulness and encourage their clients to utilize it when working to establish priority health domains. Health coaches are trained to teach breathing exercises, and also provide access to a variety of optional mobile apps that help coach someone through mindful breathing and other mindfulness exercises.

### Resources for training health coaches

Ensuring that health coaches are appropriately trained and resourced is critical for successful implementation of the M2H program. For the PMC trial, we began by providing a 1-day training in the M2H program sponsored by the US Army Medical Command. The training entailed an overview of the M2H program and philosophy, a review of the 8 health domains and their association with overall health, and how to best use the M2H wheel to prioritize and guide the conversation about health-related behaviors. Because the M2H program is centered on behavior change, MI is a critical skill to help drive success [99]. Therefore, health coaches also participated in a 2-day MI training course. Finally, an M2H toolkit for operationalizing M2H for chronic LBP was provided to the health coach. The toolkit provided a manual for the 6 steps of the M2H program for chronic LBP along with a repository of recommendations based on the scientific literature surrounding the relationships between domains of health and chronic LBP, and access to presentations from previous M2H training workshops sponsored by the US Army Medical Command.

## Discussion

The M2H program for chronic LBP was adapted from an existing program focused on a “whole person” approach to delivering medical care [20, 21]. The M2H program for chronic LBP was operationalized at a more detailed and condition-specific level than the overall M2H program in order to facilitate implementation. The tools presented in this manuscript provide the core materials and framework necessary to deliver the intervention in the PMC trial [27]. Tools will vary by setting, health system, and specific medical condition, thus efforts to implement the M2H program should make modifications as appropriate. The core elements of the creation and implementation of the M2H program developed for the PMC trial can serve as a foundation for broader application of the M2H principles.

### Patient feedback

As we have begun to implement the M2H program for persons seeking care for chronic LBP, useful feedback has been provided. Table 4 outlines several elements identified by participants as helpful, including a sense of increased accountability in making lifestyle changes facilitated by the M2H program, as well as person-centered characteristics pertaining to the health coaches.

**Table 4:** Patient-reported strengths of move to health for chronic low back pain

Major themes regarding M2H program design
Accountability (routine check-ins, weekly communication)
SMART Goal setting
General guidance Receipt of feedback/advice during the process Open-ended and patient-driven, rather than provider-directed goals
Skills and qualities of the health coach
Friendly Good listener Punctual Articulate and good at communicating Wonderful personality Genuine Patient Knowledgeable
Participants attributed some of their successes to finding joy, committing to change, incorporating activities that interested them, the use of instructional apps, and writing down thoughts and goals to reflect upon as reminders for motivation and commitment. Some participants mentioned that M2H allowed them to notice patterns in their life that related to their back pain and explore novel approaches for coping with it

### Challenges and limitations

Like with many interventions, challenges to optimal delivery exist. In our pilot of the treatment the most common and anticipated challenges included initial resistance to the overall holistic concept, dealing with pre-existing expectations, concerns about access to certain resources, clarity in identifying objective and meaningful change and progress with some lifestyle outcomes that take longer to materialize, plans for long-term stability and hand-off to other health care providers, adoption of the treatment approach at the health system level, and continuity of care across the Military Health System. At the same time, the feedback during the piloting of the program appeared to be mostly positive (Table 4). For system-wide implementation, effective use of this intervention would require an intellectual and financial investment to maintain clinicians trained to implement the model and incentivized to use it. Continued monitoring and assessment would be required to maximize evolving practices within the Military Health System.

### Future direction

The MHS strives to be a learning health system [108], where clinical research is conducted in real-world clinical settings, and the lessons learned drive changes in clinical practice and follow-on research questions [109]. Interventions like M2H require settings such as these that allow for treatments to adapt and improve. If proven successful, the M2H program will require stakeholder investment for maintenance and continued development. Further investigation will be needed to understand the long-term effectiveness (past 1 year), its value in other chronic pain conditions (musculoskeletal and otherwise), as well as assessments of cost-effectiveness. Significant investment into the resources necessary to implement this approach across the larger and diverse Military Health System will be required, as well as research to identify barriers and challenges to implementation. Additional research to assess the effectiveness of virtual health-coaching will also be of value in a setting like this, with high-operational tempo and large geographical footprint for delivering care.

### Supplementary Information

**Additional file 1.** Appendix - Implementation materials for move 2 health intervention for chronic low back pain (pdf 7313 KB)

## Data Availability

Not applicable (all materials are provided in Additional file 1: Appendix).

## References

[1] Clark LL, Taubman SB (2015). Brief report: incidence of diagnoses using ICD-9 codes specifying chronic pain (not neoplasm related) in the primary diagnostic position, active component, U.S. Armed Forces, 2007–2014. MSMR..

[2] Lew HL, Otis JD, Tun C, Kerns RD, Clark ME, Cifu DX (2009). Prevalence of chronic pain, posttraumatic stress disorder, and persistent postconcussive symptoms in OIF/OEF veterans: polytrauma clinical triad. J Rehabil Res Dev.

[3] Nahin RL (2015). Estimates of pain prevalence and severity in adults: United States, 2012. J Pain.

[4] Clark LL, Hu Z (2015). Diagnoses of low back pain, active component, U.S. Armed Forces, 2010–2014. MSMR..

[5] Schoenfeld AJ, Jiang W, Chaudhary MA, Scully RE, Koehlmoos T, Haider AH (2017). Sustained prescription opioid use among previously opioid-naive patients insured through TRICARE (2006–2014). JAMA Surg.

[6] Cohen SP, Gallagher RM, Davis SA, Griffith SR, Carragee EJ (2012). Spine-area pain in military personnel: a review of epidemiology, etiology, diagnosis, and treatment. Spine J.

[7] Alsaadi SM, McAuley JH, Hush JM, Lo S, Lin C-WC, Williams CM (2014). Poor sleep quality is strongly associated with subsequent pain intensity in patients with acute low back pain: sleep quality and pain intensity. Arthrit Rheumatol..

[8] Brooks DE, Agochukwu UF, Arrington ED, Mok JM (2013). Psychological distress in the active duty military spine patient. Mil Med.

[9] Hiebert R, Campello MA, Weiser S, Ziemke GW, Fox BA, Nordin M (2012). Predictors of short-term work-related disability among active duty US Navy personnel: a cohort study in patients with acute and subacute low back pain. Spine J.

[10] Tick H (2015). Nutrition and pain. Phys Med Rehabil Clin N Am.

[11] Vargas-Prada S, Coggon D (2015). Psychological and psychosocial determinants of musculoskeletal pain and associated disability. Best Pract Res Clin Rheumatol.

[12] Committee on Advancing Pain Research, Care, and Education, Board on Health Sciences Policy, Institute of Medicine. Relieving Pain in America: A Blueprint for Transforming Prevention, Care, Education, and Research. Washington (DC): National Academies Press; 2011. 22553896

[13] Qaseem A, Wilt TJ, McLean RM, Forciea MA, Clinical Guidelines Committee of the American College of Physicians (2017). Noninvasive treatments for acute, subacute, and chronic low back pain: a clinical practice guideline from the American College of Physicians. Ann Intern Med.

[14] Oliveira CB, Maher CG, Pinto RZ, Traeger AC, Lin C-WC, Chenot J-F (2018). Clinical practice guidelines for the management of non-specific low back pain in primary care: an updated overview. Eur Spine J.

[15] Pangarkar SS, Kang DG, Sandbrink F, Bevevino A, Tillisch K, Konitzer L (2019). VA/DoD clinical practice guideline: diagnosis and treatment of low back pain. J Gen Intern Med.

[16] Maher C, Underwood M, Buchbinder R (2017). Non-specific low back pain. Lancet.

[17] Foster NE, Anema JR, Cherkin D, Chou R, Cohen SP, Gross DP (2018). Prevention and treatment of low back pain: evidence, challenges, and promising directions. Lancet.

[18] Office of the Army Surgeon General, The. Pain Management Task Force Report. Department of Defense.

[19] Caravalho J (2015). Improving soldier health and performance by moving army medicine toward a system for health. J Strength Cond Res.

[20] Coleman AM, Hartzell MM, Oh RC, Funari TS, Rivera LO, Brown JA (2020). Improving resilience and combating burnout in US army health care teams. J Am Board Fam Med.

[21] Krejci LP, Carter K, Gaudet T (2014). Whole health: the vision and implementation of personalized, proactive, patient-driven health care for veterans. Med Care.

[22] National Center for Complementary and Integrative Health, National Institutes of Health. Complementary, alternative, or integrative health: what’s in a name? https://www.nccih.nih.gov/health/complementary-alternative-or-integrative-health-whats-in-a-name. Accessed 4 Dec 2020.

[23] Kindig DA, Asada Y, Booske B (2008). A population health framework for setting national and state health goals. JAMA.

[24] Bokhour BG, Haun JN, Hyde J, Charns M, Kligler B (2020). Transforming the veterans affairs to a whole health system of care: time for action and research. Med Care.

[25] Purcell N, Zamora K, Bertenthal D, Abadjian L, Tighe J, Seal KH (2021). How VA whole health coaching can impact veterans’ health and quality of life: a mixed-methods pilot program evaluation. Glob Adv Health Med.

[26] Kerns RD, Brandt CA, Peduzzi P (2019). NIH-DoD-VA pain management collaboratory. Pain Med.

[27] Fritz JM, Rhon DI, Teyhen DS, Kean J, Vanneman ME, Garland EL (2020). A sequential multiple-assignment randomized trial (SMART) for stepped care management of low back pain in the military health system: a trial protocol. Pain Med.

[28] Malfliet A, Coppieters I, Van Wilgen P, Kregel J, De Pauw R, Dolphens M (2017). Brain changes associated with cognitive and emotional factors in chronic pain: a systematic review. Eur J Pain.

[29] Martinez-Calderon J, Flores-Cortes M, Morales-Asencio JM, Luque-Suarez A (2020). Which psychological factors are involved in the onset and/or persistence of musculoskeletal pain? An umbrella review of systematic reviews and meta-analyses of prospective cohort studies. Clin J Pain.

[30] Haack M, Simpson N, Sethna N, Kaur S, Mullington J (2020). Sleep deficiency and chronic pain: potential underlying mechanisms and clinical implications. Neuropsychopharmacology.

[31] Song Z, Xie W, Strong JA, Berta T, Ulrich-Lai YM, Guo Q (2018). High-fat diet exacerbates postoperative pain and inflammation in a sex-dependent manner. Pain.

[32] Towery P, Guffey JS, Doerflein C, Stroup K, Saucedo S, Taylor J (2018). Chronic musculoskeletal pain and function improve with a plant-based diet. Complement Ther Med.

[33] Sharif K, Watad A, Bragazzi NL, Lichtbroun M, Amital H, Shoenfeld Y (2018). Physical activity and autoimmune diseases: get moving and manage the disease. Autoimmun Rev.

[34] Wiech K, Shriver A (2018). Cognition doesn’t only modulate pain perception; it's a central component of it. AJOB Neurosci.

[35] Ord AS, Lad SS, Shura RD, Rowland JA, Taber KH, Martindale SL (2020). Pain interference and quality of life in combat veterans: examining the roles of posttraumatic stress disorder, traumatic brain injury, and sleep quality. Rehabil Psychol.

[36] US Army Medicine. Performance Triad (P3). https://p3.amedd.army.mil/. Accessed 14 Sep 2020.

[37] McClain JJ, Lewin DS, Laposky AD, Kahle L, Berrigan D (2014). Associations between physical activity, sedentary time, sleep duration and daytime sleepiness in US adults. Prev Med.

[38] Kredlow MA, Capozzoli MC, Hearon BA, Calkins AW, Otto MW (2015). The effects of physical activity on sleep: a meta-analytic review. J Behav Med.

[39] Doherty R, Madigan S, Warrington G, Ellis J (2019). Sleep and nutrition interactions: implications for athletes. Nutrients.

[40] Walker MP (2009). The role of sleep in cognition and emotion. Ann N Y Acad Sci.

[41] Ben Simon E, Vallat R, Barnes CM, Walker MP (2020). Sleep loss and the socio-emotional brain. Trends Cogn Sci.

[42] Kelly GA, Blake C, Power CK, O’keeffe D, Fullen BM (2011). The association between chronic low back pain and sleep: a systematic review. Clin J Pain.

[43] Artner J, Cakir B, Spiekermann J-A, Kurz S, Leucht F, Reichel H (2013). Prevalence of sleep deprivation in patients with chronic neck and back pain: a retrospective evaluation of 1016 patients. J Pain Res.

[44] Tang NKY, Wright KJ, Salkovskis PM (2007). Prevalence and correlates of clinical insomnia co-occurring with chronic back pain. J Sleep Res.

[45] Alsaadi SM, McAuley JH, Hush JM, Maher CG (2011). Prevalence of sleep disturbance in patients with low back pain. Eur Spine J.

[46] Marin R, Cyhan T, Miklos W (2006). Sleep disturbance in patients with chronic low back pain. Am J Phys Med Rehabil.

[47] Sivertsen B, Lallukka T, Petrie KJ, Steingrímsdóttir ÓA, Stubhaug A, Nielsen CS (2015). Sleep and pain sensitivity in adults. Pain.

[48] Haack M, Sanchez E, Mullington JM (2007). Elevated inflammatory markers in response to prolonged sleep restriction are associated with increased pain experience in healthy volunteers. Sleep.

[49] Irwin MR, Olmstead R, Carroll JE (2016). Sleep disturbance, sleep duration, and inflammation: a systematic review and meta-analysis of cohort studies and experimental sleep deprivation. Biol Psychiatry.

[50] Agmon M, Armon G (2014). Increased insomnia symptoms predict the onset of back pain among employed adults. PLoS ONE.

[51] Skarpsno ES, Mork PJ, Nilsen TIL, Nordstoga AL (2020). Influence of sleep problems and co-occurring musculoskeletal pain on long-term prognosis of chronic low back pain: the HUNT Study. J Epidemiol Community Health.

[52] Army Public Health Center (APHC). DoD Health of the Force Report 2015. Army Office of the Surgeon General; 2015. https://api.army.mil/e2/c/downloads/419337.pdf.

[53] de Campos TF, Maher CG, Fuller JT, Steffens D, Attwell S, Hancock MJ (2020). Prevention strategies to reduce future impact of low back pain: a systematic review and meta-analysis. Br J Sports Med.

[54] Marley J, Tully MA, Porter-Armstrong A, Bunting B, O’Hanlon J, Atkins L (2017). The effectiveness of interventions aimed at increasing physical activity in adults with persistent musculoskeletal pain: a systematic review and meta-analysis. BMC Musculoskelet Disord.

[55] Geneen LJ, Moore RA, Clarke C, Martin D, Colvin LA, Smith BH (2017). Physical activity and exercise for chronic pain in adults: an overview of cochrane reviews. Cochrane Database Syst Rev.

[56] Malfliet A, Ickmans K, Huysmans E, Coppieters I, Willaert W, Van Bogaert W (2019). Best evidence rehabilitation for chronic pain part 3: low back pain. J Clin Med Res.

[57] Lin I, Wiles L, Waller R, Goucke R, Nagree Y, Gibberd M (2020). What does best practice care for musculoskeletal pain look like? Eleven consistent recommendations from high-quality clinical practice guidelines: systematic review. Br J Sports Med.

[58] Meadows SO, Engel CC, Collins RL, Beckman R, Cefalu M, Hawes-Dawson J, et al. 2015 HRBS Final Report 508 Compliant. Department of Defense; 2018. https://www.health.mil/Reference-Center/Reports/2018/06/21/2015-HRBS-Final-Report.

[59] Global Recommendations on Physical Activity for Health. World Health Organization; 2010. https://apps.who.int/iris/bitstream/handle/10665/44399/9789241599979_eng.pdf. 26180873

[60] Boutevillain L, Dupeyron A, Rouch C, Richard E, Coudeyre E (2017). Facilitators and barriers to physical activity in people with chronic low back pain: a qualitative study. PLoS ONE.

[61] Karlsson L, Gerdle B, Takala E-P, Andersson G, Larsson B (2018). Experiences and attitudes about physical activity and exercise in patients with chronic pain: a qualitative interview study. J Pain Res.

[62] Marshall PWM, Schabrun S, Knox MF (2017). Physical activity and the mediating effect of fear, depression, anxiety, and catastrophizing on pain related disability in people with chronic low back pain. PLoS ONE.

[63] Meleger AL, Froude CK, Walker J (2014). Nutrition and eating behavior in patients with chronic pain receiving long-term opioid therapy. PM R.

[64] Brain K, Burrows TL, Rollo ME, Chai LK, Clarke ED, Hayes C (2019). A systematic review and meta-analysis of nutrition interventions for chronic noncancer pain. J Hum Nutr Diet.

[65] Walsh TP, Arnold JB, Evans AM, Yaxley A, Damarell RA, Shanahan EM (2018). The association between body fat and musculoskeletal pain: a systematic review and meta-analysis. BMC Musculoskelet Disord.

[66] Nijs J, Tumkaya Yilmaz S, Elma Ö, Tatta J, Mullie P, Vanderweeën L (2020). Nutritional intervention in chronic pain: an innovative way of targeting central nervous system sensitization?. Expert Opin Ther Targets.

[67] Guo R, Chen L-H, Xing C, Liu T (2019). Pain regulation by gut microbiota: molecular mechanisms and therapeutic potential. Br J Anaesth.

[68] Elma Ö, Yilmaz ST, Deliens T, Coppieters I, Clarys P, Nijs J (2020). Do nutritional factors interact with chronic musculoskeletal pain? A systematic review. J Clin Med Res.

[69] Elma Ö, Yilmaz ST, Deliens T, Clarys P, Nijs J, Coppieters I (2020). Chronic musculoskeletal pain and nutrition: where are we and where are we heading?. PM R.

[70] Bear T, Philipp M, Hill S, Mündel T (2016). A preliminary study on how hypohydration affects pain perception. Psychophysiology.

[71] Rondanelli M, Faliva MA, Miccono A, Naso M, Nichetti M, Riva A (2018). Food pyramid for subjects with chronic pain: foods and dietary constituents as anti-inflammatory and antioxidant agents. Nutr Res Rev.

[72] Gentili C, Rickardsson J, Zetterqvist V, Simons LE, Lekander M, Wicksell RK (2019). Psychological flexibility as a resilience factor in individuals with chronic pain. Front Psychol.

[73] Bushnell MC, Ceko M, Low LA (2013). Cognitive and emotional control of pain and its disruption in chronic pain. Nat Rev Neurosci.

[74] Vachon-Presseau E, Centeno MV, Ren W, Berger SE, Tétreault P, Ghantous M (2016). The emotional brain as a predictor and amplifier of chronic pain. J Dent Res.

[75] Ferreira-Valente A, Sharma S, Torres S, Smothers Z, Pais-Ribeiro J, Abbott JH (2019). Does religiosity/spirituality play a role in function, pain-related beliefs, and coping in patients with chronic pain? A systematic review. J Relig Health.

[76] Day MA, Ward LC, Thorn BE, Burns J, Ehde DM, Barnier AJ (2020). Mechanisms of mindfulness meditation, cognitive therapy, and mindfulness-based cognitive therapy for chronic low back pain. Clin J Pain.

[77] Veehof MM, Trompetter HR, Bohlmeijer ET, Schreurs KMG (2016). Acceptance- and mindfulness-based interventions for the treatment of chronic pain: a meta-analytic review. Cogn Behav Ther.

[78] Shiri R, Karppinen J, Leino-Arjas P, Solovieva S, Viikari-Juntura E (2010). The association between smoking and low back pain: a meta-analysis. Am J Med.

[79] Helmhout PH, Staal JB, Heymans MW, Harts CC, Hendriks EJM, de Bie RA (2010). Prognostic factors for perceived recovery or functional improvement in non-specific low back pain: secondary analyses of three randomized clinical trials. Eur Spine J.

[80] Steenstra IA, Verbeek JH, Heymans MW, Bongers PM (2005). Prognostic factors for duration of sick leave in patients sick listed with acute low back pain: a systematic review of the literature. Occup Environ Med.

[81] Bender JL, Radhakrishnan A, Diorio C, Englesakis M, Jadad AR (2011). Can pain be managed through the Internet? A systematic review of randomized controlled trials. Pain.

[82] Robles TF (2014). Marital quality and health: implications for marriage in the 21st century. Curr Dir Psychol Sci.

[83] Jamison RN, Virts KL (1990). The influence of family support on chronic pain. Behav Res Ther.

[84] Kiecolt-Glaser JK, Wilson SJ (2017). Lovesick: how couples’ relationships influence health. Annu Rev Clin Psychol.

[85] Martire LM, Keefe FJ, Schulz R, Parris Stephens MA, Mogle JA (2013). The impact of daily arthritis pain on spouse sleep. Pain.

[86] Jackson SE, Steptoe A, Wardle J (2015). The influence of partner’s behavior on health behavior change: the English Longitudinal Study of Ageing. JAMA Intern Med.

[87] NHLBI Obesity Education Initiative Expert Panel on the Identification, Evaluation, Treatment of Obesity. Executive Summary. National Heart, Lung, and Blood Institute; 1998.

[88] Godin G, Shephard RJ (1985). A simple method to assess exercise behavior in the community. Can J Appl Sport Sci.

[89] Buysse DJ, Yu L, Moul DE, Germain A, Stover A, Dodds NE (2010). Development and validation of patient-reported outcome measures for sleep disturbance and sleep-related impairments. Sleep.

[90] Nayfe R, Chansard M, Hynan LS, Mortensen EM, Annaswamy T, Fraenkel L (2020). Comparison of patient-reported outcomes measurement information system and legacy instruments in multiple domains among older veterans with chronic back pain. BMC Musculoskelet Disord.

[91] Gruber-Baldini AL, Velozo C, Romero S, Shulman LM (2017). Validation of the PROMIS^®^ measures of self-efficacy for managing chronic conditions. Qual Life Res.

[92] Cella D, Choi SW, Condon DM, Schalet B, Hays RD, Rothrock NE (2019). PROMIS^®^ adult health profiles: efficient short-form measures of seven health domains. Value Health.

[93] Krebs EE, Lorenz KA, Bair MJ, Damush TM, Wu J, Sutherland JM (2009). Development and initial validation of the PEG, a three-item scale assessing pain intensity and interference. J Gen Intern Med.

[94] Khalifeh JM, Dibble CF, Hawasli AH, Ray WZ (2019). Patient-reported outcomes measurement information system physical function and pain interference in spine surgery. J Neurosurg Spine.

[95] Buckenmaier CC, Galloway KT, Polomano RC, McDuffie M, Kwon N, Gallagher RM (2013). Preliminary validation of the defense and veterans pain rating scale (DVPRS) in a military population. Pain Med.

[96] Hill JC, Dunn KM, Lewis M, Mullis R, Main CJ, Foster NE (2008). A primary care back pain screening tool: identifying patient subgroups for initial treatment. Arthritis Rheum.

[97] EuroQol Group (1990). EuroQol—a new facility for the measurement of health-related quality of life. Health Policy.

[98] Herdman M, Gudex C, Lloyd A, Janssen M, Kind P, Parkin D (2011). Development and preliminary testing of the new five-level version of EQ-5D (EQ-5D-5L). Qual Life Res.

[99] Frost H, Campbell P, Maxwell M, O’Carroll RE, Dombrowski SU, Williams B (2018). Effectiveness of motivational interviewing on adult behaviour change in health and social care settings: a systematic review of reviews. PLoS ONE.

[100] Harman K, Macrae M, Vallis M, Bassett R (2014). Working with people to make changes: a behavioural change approach used in chronic low back pain rehabilitation. Physiother Can.

[101] Waddell G, Newton M, Henderson I, Somerville D, Main CJ (1993). A fear-avoidance beliefs questionnaire (FABQ) and the role of fear-avoidance beliefs in chronic low back pain and disability. Pain.

[102] Amireault S, Godin G (2015). The Godin-Shephard leisure-time physical activity questionnaire: validity evidence supporting its use for classifying healthy adults into active and insufficiently active categories. Percept Mot Skills.

[103] Purvis DL, Lentino CV, Jackson TK, Murphy KJ, Deuster PA. Nutrition as a component of the performance triad: how healthy eating behaviors contribute to soldier performance and military readiness. US Army Med Dep J. 2013;66–78. 24146244

[104] Gardner T, Refshauge K, McAuley J, Goodall S, Hübscher M, Smith L (2015). Patient led goal setting in chronic low back pain-What goals are important to the patient and are they aligned to what we measure?. Patient Educ Couns.

[105] Bandura A (1977). Self-efficacy: toward a unifying theory of behavioral change. Psychol Rev.

[106] Kwasnicka D, Presseau J, White M, Sniehotta FF (2013). Does planning how to cope with anticipated barriers facilitate health-related behaviour change? A systematic review. Health Psychol Rev.

[107] Hanley AW, Gililland J, Garland EL (2021). To be mindful of the breath or pain: comparing two brief preoperative mindfulness techniques for total joint arthroplasty patients. J Consult Clin Psychol.

[108] Rasmussen TE, Kellermann AL, Rauch TM (2020). A primer on the military health system’s approach to medical research and development. Acad Med.

[109] Lewis RJ (2016). The pragmatic clinical trial in a learning health care system. Clin Trials.

