# Correction: move to health-a holistic approach to the management of chronic low back pain: an intervention and implementation protocol developed for a pragmatic clinical trial

**DOI:** 10.1186/s12967-024-05343-z

**Published:** 2024-06-17

**Authors:** Daniel I. Rhon, Julie M. Fritz, Tina A. Greenlee, Katie E. Dry, Rachel J. Mayhew, Mary C. Laugesen, Edita Dragusin, Deydre S. Teyhen

**Affiliations:** 1 Department of Rehabilitation Medicine, Brooke Army Medical Center, JBSA Fort Sam Houston, 3551 Roger Brooke Drive, San Antonio, TX 78234 USA; 2 Department of Rehabilitation Medicine, Uniformed Services University of Health Sciences, Bethesda, MD USA; 3 University of Utah, Salt Lake City, UT USA; 4 Walter Reed Army Institute of Research, Silver Spring, MD USA

**Journal of Translational Medicine (2021) 19:357**

10.1186/s12967-021-03013-y

Following publication of the original article [1], we have been notified that article body text contains incorrectly published link.

It is now:

Step 2: personal health inventory.

Following review of health history, the person with LBP views an introductory video linking M2H to LBP (www.vimeo.com/XXXXX) and then the PHI (SA1) is completed.

It should be as per below:

Step 2: personal health inventory.

Following review of health history, the person with LBP views an introductory video linking M2H to LBP (https://vimeo.com/498500657) and then the PHI (SA1) is completed.
