# Supplementary material for: Move to health-a holistic approach to the management of chronic low back pain: an intervention and implementation protocol developed for a pragmatic clinical trial
Source: J Transl Med. 2021 Aug 18;19:357. doi: 10.1186/s12967-021-03013-y (PMC8371880; doi:10.1186/s12967-021-03013-y)
Supplement: Supplementary file 1 — Additional file 1. Appendix - Implementation materials for move 2 health intervention for chronic low back pain (pdf 7313 KB) [file 12967_2021_3013_MOESM1_ESM.pdf]

Supplementary Appendix (SA)
Implementation Materials for Move 2 Health Intervention for Chronic Low Back Pain

Page(s)	
1	Table of Contents
2	Acronyms
3-6	1.1: Personal Health Inventory
7-8	1.2: Advanced Sleep Screen Algorithm
9-11	1.3: M2H Summary Report
12-16	1.4: Health Coach Note Templates
17	1.5: S.M.A.R.T Goal Setting Worksheet
18-34	1.6: Educational Handouts
35-40	1.7: Mobile Apps
41-42	1.8: Additional website resources

Acronyms

AASM	American Academy of Sleep Medicine
APRT	Army Physical Readiness Test/Training
AWC	Army Wellness Center
BH	Behavioral Health
BMI	Body Mass Index
BP	Blood Pressure
CBTi	Cognitive Behavioral Therapy for Insomnia
CDC	Centers for Disease Control and Prevention
COVID	corona (CO), virus (VI), disease (D)
DoD	Department of Defense
EQ-5D-5L	European Quality of Life Questionnaire - 5 Dimensions - 5 Levels
EMR	Electronic Medical Record
ESS	Epworth Sleepiness Scale
FABQ-PA	Fear-avoidance Beliefs Questionnaire - Physical Activity Subscale
FABQ-W	Fear-avoidance Beliefs Questionnaire - Work Subscale
GROC	Global Rating of Change scale
HPRC	Human Performance Resource Center
ISI	Insomnia Sleep Index
M2H	Move to Health; Move 2 Health
MWR	Morale Welfare and Recreation
NIH	National Institutes of Health
PCM	Primary Care Manager
PC-PTSD-5	Primary Care Post-traumatic Stress Disorder Screen for Diagnostic and Statistical Manual of Mental Disorders, Fifth Edition
PEG-3	Pain Average, Enjoyment of Life, & General Activity
PFA	Physical Fitness Assessment
PHQ-9	Patient Health Questionnaire - 9
PROMIS	Patient Reported Outcomes Measurement Information System
PSQI	Pittsburgh Sleep Quality Index
PTSD	Post-traumatic Stress Disorder
SBST	Subgroups for Targeted Treatment (STarT) Back Screening Tool
SHARP	Sexual Harassment/Assault Response and Prevention
SMART	Specific; Measurable; Attainable; Realistic; Time-bound
STOPBANG	Snoring. Tiredness; Observed you stop breathing; blood Pressure; BMI; Age; Neck circumference > 40cm (>15.7”); Gender male
T2	Army Research Laboratory Technology Transfer
TBI	Traumatic Brain Injury
USDA	United States Department of Agriculture
VA	Veterans Affairs

Move to Health: Changing the Conversation in Army Medicine

MyStory: Personal Health Inventory

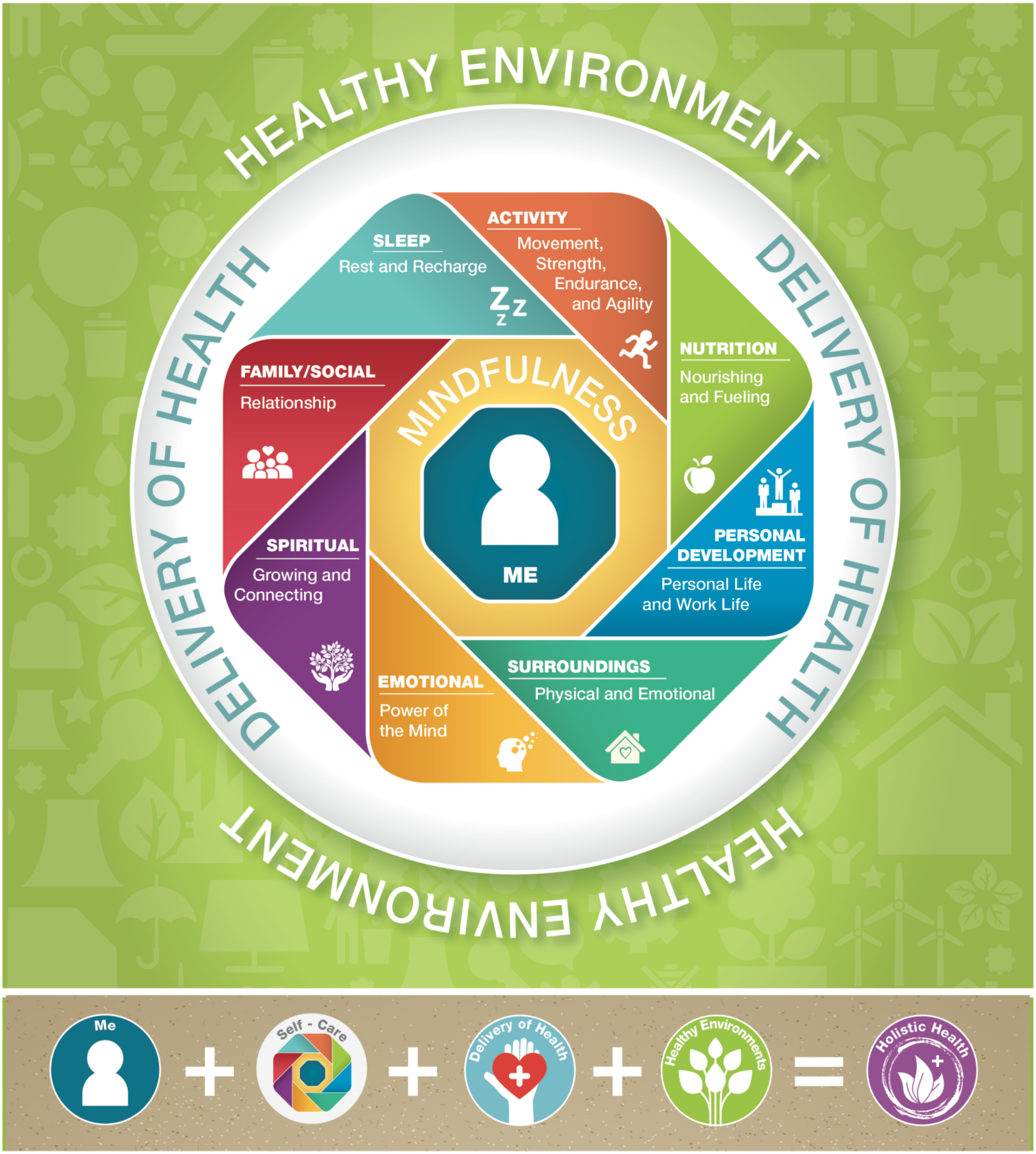

My Story: Personal Health Inventory

Your Personal Health Inventory

1. What REALLY matters to you in your life?

2. What brings you a sense of joy and happiness?

3. On the following scale from 1-5, with 1 being miserable and 5 being great, circle where you feel you are on the scale.

Physical Well-Being:

1 2 3 4 5

Miserable **Great**

Mental/Emotional Well-Being:

1 2 3 4 5

Miserable **Great**

Life: How is it to live your day-to-day life?

1 2 3 4 5

Miserable **Great**

Where You Are And Where You'd Like To Be

For each area below, consider where you are now and where you would like to be. All the areas are important. In the "Where you are" box, briefly write the reasons you chose your number. In the "Where you want to be" box, write down some changes that might make this area better for you. Some areas are strongly connected to other areas, so you may notice some of your answers seem the same. Try to fill out as many areas as you can. You do not have to write in every area or in all the areas at one time. You might want to start with the easier ones and come back to the harder ones. It is OK just to circle the numbers.

Sleep: *"Rest and Recharge"* Getting enough sleep, rest, and relaxation.

Where you are:

Rate yourself on a scale of 1 (low) to 5 (high)

1 2 3 4 5

What are the reasons you choose this number?

Where would you like to be?

1 2 3 4 5

What changes could you make to help you get there?

Activity: "*Movement, Strength, Endurance and Agility"* Includes movement and physical activities like walking, dancing, gardening, sports, lifting weights, yoga, cycling, swimming, and working out in a gym.

Where you are:

Rate yourself on a scale of 1 (low) to 5 (high)

1 2 3 4 5

What are the reasons you choose this number?

Where would you like to be?

1 2 3 4 5

What changes could you make to help you get there?

Nutrition: *"Nourishing and Fueling"* Eating healthy, balanced meals with plenty of fruits and vegetables each day. Drinking enough water and limiting sodas, sweetened drinks, and alcohol.

Where you are:
Rate yourself on a scale of 1 (low) to 5 (high)
1 2 3 4 5

What are the reasons you choose this number?

Where would you like to be?
1 2 3 4 5

What changes could you make to help you get there?

Personal Development: *"Personal life and Work life"* Learning and growing. Developing abilities and talents. Balancing responsibilities where you live, volunteer, and work.

Where you are:
Rate yourself on a scale of 1 (low) to 5 (high)
1 2 3 4 5

What are the reasons you choose this number?

Where would you like to be?
1 2 3 4 5

What changes could you make to help you get there?

Family/Social: *"Relationships"* Feeling listened to and connected to people you love and care about. The quality of your communication with family, friends, and people you work with.

Where you are:
Rate yourself on a scale of 1 (low) to 5 (high)
1 2 3 4 5

What are the reasons you choose this number?

Where would you like to be?
1 2 3 4 5

What changes could you make to help you get there?

Spiritual: *"Growing and Connecting"* Having a sense of purpose and meaning in your life. Feeling connected to something larger than yourself. Finding strength in difficult times.

Where you are:
Rate yourself on a scale of 1 (low) to 5 (high)
1 2 3 4 5

What are the reasons you choose this number?

Where would you like to be?
1 2 3 4 5

What changes could you make to help you get there?

Surroundings: *Physical and Emotional"* Feeling safe. Having comfortable, healthy spaces where you work and live. The quality of the lighting, color, air, and water. Decreasing unpleasant clutter, noises, and smells.

Where you are:
Rate yourself on a scale of 1 (low) to 5 (high)
1 2 3 4 5

What are the reasons you choose this number?

____
____
____
____

Where would you like to be?
1 2 3 4 5

What changes could you make to help you get there?

____
____
____
____

Emotional: *"Power of the Mind"* Tapping into the power of your mind to heal and cope. Using mid-body techniques like relaxation, breathing, or guided imagery.

Where you are:
Rate yourself on a scale of 1 (low) to 5 (high)
1 2 3 4 5

What are the reasons you choose this number?

____
____
____
____

Where would you like to be?
1 2 3 4 5

What changes could you make to help you get there?

____
____
____
____

Professional Care

Prevention: On a scale of 1-5, circle the number that best describes how up to date you are on your preventive care such as influenza vaccine, cholesterol check, cancer screening, and dental care.

1	2	3	4	5
**Not at all**	**A little bit**	**Somewhat**	**Quite a bit**	**Very much**

Clinical Care: If you are working with a healthcare professional, on a scale of 1-5, circle the number that best describes how well you understand your health problems, the treatment plan, and your role in your health.

- ○ I am not working with a healthcare professional

1	2	3	4	5
**Not at all**	**A little bit**	**Somewhat**	**Quite a bit**	**Very much**

Reflections

1. Now that you have thought about all of these areas, what is your vision of your best possible health? What would your life look like? What kind of activities would you be doing?

____
____
____

2. Are there any areas you would like to work on? Where might you start?

____
____
____

Advanced Sleep Screen

Q1:
How many hours of sleep would you estimate that you currently get per night (or in a 24 hour period)?

≥ 9 hrs → Administer PHQ-9[1,2]

7-8 hrs → Go to Q2

5-6 hrs → Go to Q5 AND **Commence Sleep Education**

< 5 hrs → Go to Q5 AND Administer ISI[3,4], ESS[5,6,7,8], STOPBANG[9,10], PSQI[11] AND **Commence Sleep Education**

PHQ-9 Positive Score (4 ✓ in shaded domains)
Refer to BH
PHQ-9 Negative Score → Go to Q5 AND
Commence Sleep Education

Q2: Are you satisfied with your sleep quality?

No → Go to Q3 and Q5

Yes → Go to Q4 and Q5

Q3: Do you feel that your sleep quality is affecting your day time functioning?

No → No Referral. Reassure patient sleep seems on track and suggest another domain of the wheel.

Yes → Go to Q5 AND Administer ISI, ESS, STOPBANG, PSQI

ISI Score 0-14
CBT-i/Sleep Ed.
ISI Score 15-28
CBT-i/Sleep Ed.
Refer to BH

ESS Score 0-10
Sleep Ed.
ESS Score 11-24
CBT-i/Sleep Ed.
Refer to BH/Sleep Clinic

Q4: The following best describes my sleep quality:

I get restful sleep and don't have any issues I know about. I may have snoring or an occasional dream/nightmare but it doesn't interfere with my sleep.
→ No Referral. Reassure patient sleep seems on track and suggest another domain of the wheel.

I snore and don't get restful sleep.
→ Go to Q5 AND Administer STOPBANG

I have dreams/nightmares that interfere with my sleep quality.
→ Go to Q5 AND Administer PC-PTSD-5[12]

I don't get restful sleep. → Go to Q5 AND Administer ISI, ESS

STOPBANG 3+ "yes"
Sleep Ed.
Refer to Sleep Clinic

PSQI global score > 5
CBT-i/Sleep Ed.

PC-PTSD-5 3+ "yes"
CBT-i/Sleep Ed.
Refer to Sleep Clinic

Q5: Caffeine is found in coffee, tea, sodas, some kinds of gum and energy drinks.

I don't use any products containing caffeine. → No further action

I rarely use any products containing caffeine. → No further action

I use caffeine every day, in the morning to wake up and sometimes during the day if I need a boost, but I don't rely on it too much. → **Commence Sleep Education/Consider Nutrition Domain**

I use caffeine every day, and I probably rely too much on it, but I'm not interested in any help at this time.
→ **Commence Sleep Education/Consider Nutrition Domain**

I use caffeine every day, and I probably rely too much on it, and I'd like to talk to someone about reducing my reliance on caffeine. → **Commence Sleep Education/Nutrition Referral**

Note: Adapted version

References:

1 Kroenke K, Spitzer RL, Williams JB. The PHQ-9: validity of a brief depression severity measure. J Gen Intern Med. 2001 Sep;16(9):606-13. doi: 10.1046/j.1525-1497.2001.016009606.x. PMID: 11556941; PMCID: PMC1495268.

2 Kroenke, Kurt, and Robert L. Spitzer. 2002. "The PHQ-9: A New Depression Diagnostic and Severity Measure." *Psychiatric Annals* 32 (9): 509–15.

3 Bastien CH, Vallières A, Morin CM. Validation of the Insomnia Severity Index as an outcome measure for insomnia research. Sleep Med. 2001 Jul;2(4):297-307. doi: 10.1016/s1389-9457(00)00065-4. PMID: 11438246.

4 Morin, Charles M., Geneviève Belleville, Lynda Bélanger, and Hans Ivers. 2011. "The Insomnia Severity Index: Psychometric Indicators to Detect Insomnia Cases and Evaluate Treatment Response." *Sleep* 34 (5): 601–8.

5 Johns MW. A new method for measuring daytime sleepiness: the Epworth Sleepiness Scale. Sleep, 1991; 14: 50-55.

6 "Epworth Sleepiness Scale – The Official Website of the Epworth Sleepiness Scale (ESS & ESS-CHAD)." n.d. Accessed March 29, 2019. https://epworthsleepinessscale.com/.

7 Lundt, Leslie. 2005. "Use of the Epworth Sleepiness Scale to Evaluate the Symptom of Excessive Sleepiness in Major Depressive Disorder." *General Hospital Psychiatry* 27 (2): 146–48.

8 Gander, Philippa H., Nathaniel S. Marshall, Ricci Harris, and Papaarangi Reid. 2005. "The Epworth Sleepiness Scale: Influence of Age, Ethnicity, and Socioeconomic Deprivation. Epworth Sleepiness Scores of Adults in New Zealand." *Sleep* 28 (2): 249–53.

9 Chung, Frances, Hairil R. Abdullah, and Pu Liao. 2016. "STOP-Bang Questionnaire." *Chest* 149 (3): 631–38.

10 Nagappa, Mahesh, Pu Liao, Jean Wong, Dennis Auckley, Satya Krishna Ramachandran, Stavros Memtsoudis, Babak Mokhlesi, and Frances Chung. 2015. "Validation of the STOP-Bang Questionnaire as a Screening Tool for Obstructive Sleep Apnea among Different Populations: A Systematic Review and Meta-Analysis." *PloS One* 10 (12): e0143697.

11 Buysse DJ, Reynolds CF, Monk TH, Berman SR, Kupfer DJ: The Pittsburgh Sleep Quality Index: A new instrument for psychiatric practice and research. Psychiatry Research 28:193-213, 1989.

12 Prins, A., Bovin, M. J., Kimerling, R., Kaloupek, D. G, Marx, B. P., Pless Kaiser, A., & Schnurr, P. P. (2015). Primary Care PTSD Screen for DSM-5 (PC-PTSD-5) [Measurement instrument]. Available from https://www.ptsd.va.gov URL: https://www.ptsd.va.gov/professional/ assessment/screens/pc-ptsd.asp

M2H Summary Report

Please indicate if you do or don't have this common health problem and if it limits your daily activity:

	I don't have this problem	I have had this problem in the past	I currently have this problem and it doesn't limit my daily activity	I currently have this problem and it limits my daily activity
Tobacco/Nicotine use in past year?:	O	O	O	O
Drug/Alcohol Problem:	O	O	O	O
Anxiety:	O	O	O	O
PTSD:	O	O	O	O
Depression:	O	O	O	O
TBI/Concussion:	O	O	O	O
Sleep Disorder:	O	O	O	O
Diabetes:	O	O	O	O
High Blood Pressure:	O	O	O	O

BMI: _______

< 18.5 = underweight; 25 to 29.9 = overweight; ≥ 30 = obese

Keele STarT Back

SBST Total Score: ___________ SBST Psychosocial Subscale Score: ___________

This is a ________________ RISK participant!

Total score ≤ 3 points = low risk; Total score ≥ 4 points (≤ 3 points on Qs 5-9 = medium risk; 4+ points on Qs 5-9 = high risk)

Patient Reported Outcomes Measurement Information System (PROMIS)

PROMIS - Pain Interference: ___________ PROMIS - Depression: ___________

PROMIS - Physical Function: ___________ PROMIS - Anxiety: ___________

PROMIS - Sleep Disturbance: ___________

All PROMIS scores are reported on a metric with a score of 50 points aligning with the general population mean with a standard deviation of 10. HIGHER SCORES (50+) indicate GREATER pain interference, sleep disturbance, depression, anxiety and physical function (POSITIVE) than the general population. **Review individual items of the PROMIS domains to gain better perspective of why the patient chose those answers.**

Pain Average, Enjoyment of Life, & General Activity (PEG)- 3

PEG-3 Total Average: ___________ Pain Interferes with Enjoyment of Life: ___________

Pain on Average: ___________ Pain Interferes with General Activity: ___________

3 PEG Score: Average of Sum Total

Pain: 0 (No pain) <> 10 (Pain as bad as you can imagine)

Pain interferes with Enjoyment: 0 (Doesn't interfere) <> 10 (Completely interferes)

Pain interferes with General Activity: 0 (Doesn't interfere) <> 10 (Completely interferes)

EuroQol – 5 Dimensions (EQ-5D)

EQ-5D Mobility: ___________ EQ-5D Pain/Discomfort: ___________

EQ-5D Self-Care: ___________ EQ-5D Anxiety/Depression: ___________

EQ-5D Usual Activities: ___________

EQ-5D Health Report as of TODAY: 0 (Worst Health) <> 100 (Best Health): ___________

1: Indicating NO Problems
3: Indicating MODERATE Problems
5: Indicating UNABLE TO or EXTREME Problems

NUTRITION

Healthy Eating Score – 5: ___________

20-25: More likely to eat breakfast, have a healthy BMI, drink at least 7 servings of water/day or more, have a healthy weight size, and eat an exercise recovery snack
17-19: Third quartile
13-16: Second quartile
0-12: More likely to drink soda and binge drink alcohol; Refer to dietetics

ACTIVITY

Fear-Avoidance Beliefs Questionnaire

FABQ-Physical Activity Score: ___________ FABQ-Work Score: ___________

>15: Physical Activity Subscale - High Fear Avoidance
34+: Work Subscale - High Fear Avoidance

Godin Leisure-Time Exercise Questionnaire: ___________

24+: Active
14-23: Moderately Active
< 14: Insufficiently Active/Sedentary

SLEEP

Epworth Sleepiness Scale: ___________

0-10: No action needed
11-12: Possibly Refer
13-24: Refer to Sleep Clinic/Behavioral Health

Insomnia Severity Index: ___________

0-7: No Clinically Significant Insomnia; Sleep Ed
8-14: Sub-Threshold Insomnia; Sleep Ed, Consider Referral to Sleep Clinic/BH
15-21: Moderate Severity Clinical Insomnia; Refer to Sleep Clinic/Behavioral Health
22-28: Severe Clinical Insomnia; Refer to Sleep Clinic/Behavioral Health

M2H Summary Report

STOPBANG – Sleep Apnea: ___________

0-2: Low Risk; No action needed
3-4: Intermediate Risk; Sleep Ed. Referral to Sleep Clinic/BH
5-8: High Risk; Referral to Sleep Clinic/BH

Patient Health Questionnaire-9: ___________

1-4: None - Minimal Depression: No action needed
5-9: Mild: Be mindful
10-14: Moderate: Referral to Behavioral Health
15-19: Moderately Severe: Immediate Action Per Clinic Policy
20-27: Severe Depression: Immediate Action Per Clinic Policy

Pittsburgh Sleep Quality Index: ___________
PSQI Score Interpretation: [Good OR Poor] Sleep Quality

0: Minimum Score (better)   21: Maximum Score (worse)

Pittsburgh Sleep Quality Index: Sub Domains

PSQI Sleep Quality: ___________
PSQI Sleep Latency: ___________
PSQI Sleep Duration: ___________
PSQI Sleep Efficiency: ___________
PSQI Sleep Disturbance: ___________
PSQI Use of Sleep Medication: ___________
PSQI Daytime Dysfunction: ___________

0: Minimum Score (better)   3: Maximum Score (worse)

Primary Care-Post Traumatic Stress Disorder 5: ___________

0-2: No action needed
3: May have PTSD; Consider Referral to Sleep Clinic/BH
4+: Likely has PTSD; Refer to Sleep Clinic/BH; Notify PCM

Sleep Referral Determination

Includes any of the recommendations listed below, based upon Advanced Sleep Screen:

- No Referral. Reassure the patient that their sleep seems on track and suggest another component of the wheel.
- Commence sleep education which includes domain specific handout, video, and discussing other resources.
- Please consider dietetics referral due to moderate caffeine intake.
  Please put in a dietetics referral due to high caffeine intake.
- Please refer to behavioral health/sleep clinic (+ CBTi app download). Please refer to behavioral health for depression.

M2H Coach Initial

PARTICIPANT

Active SMART Goal ________________________________

Active SMART Goal Domain
- ◯ Sleep
- ◯ Activity
- ◯ Nutrition
- ◯ Intrinsic
- ◯ Extrinsic

What type of barrier(s) and solution(s) to the given barrier(s) are anticipated by the participant? ________________________________

Appointment Duration (Minutes) ________________________________

Actions and Plans for Active SMART Goal?
- ☐ Emailed participant new domain information
- ☐ Submitted EMR consult/referral
- ☐ Submitted M2H referral (non-EMR)
- ☐ Scheduled M2H follow-up session
- ☐ Introduced a military resource (i.e. class, website, app, phone number, literature)
- ☐ Introduced a community resource (i.e. class, website, app, phone number, literature)
- ☐ Other

List the military resource(s) introduced:

List the community resource(s) introduced:

Other Action:

COACH

Session Type:
- ◯ In-person
- ◯ Voice Only
- ◯ Video
- ◯ Other

Explain "Other":

As the M2H coach, how would YOU gauge your participant's % investment in this intervention?

0% ▭▭▭▭▭▭▭▭▭▭▭▭▭▭▭▭▭▭▭▭ 100%

(Place a mark on the scale above)

Coach Notes:
*Write any reminders here that you want to reference in the future follow up form!
*What challenges did you have?
*Participant's % confidence in achieving goal?
*What worked well for you?
*Details to remember about this participant; information for future interactions to help engage:
*Tools that proved to be effective today:
*Other:

Motivational Interviewing Self-Assessment: To what degree did you implement these components during your interaction today?

	0 Not at all	1	2	3	4	5	6	7	8	9	10 Grand Slam
Open Ended Questions	◯	◯	◯	◯	◯	◯	◯	◯	◯	◯	◯
Affirmations	◯	◯	◯	◯	◯	◯	◯	◯	◯	◯	◯
Reflective Listening (Change Talk)	◯	◯	◯	◯	◯	◯	◯	◯	◯	◯	◯
Summaries	◯	◯	◯	◯	◯	◯	◯	◯	◯	◯	◯
Suppressing Righting Reflex	◯	◯	◯	◯	◯	◯	◯	◯	◯	◯	◯
Engage	◯	◯	◯	◯	◯	◯	◯	◯	◯	◯	◯
Focus	◯	◯	◯	◯	◯	◯	◯	◯	◯	◯	◯
Evoke	◯	◯	◯	◯	◯	◯	◯	◯	◯	◯	◯
Plan	◯	◯	◯	◯	◯	◯	◯	◯	◯	◯	◯

M2H Coach Follow-up

M2H Follow Up #1 – Date: __________

Prior M2H Session Coach Notes: ___________

Prior M2H Session Solutions to Perceived Barriers: ___________

SMART Goal #1

SMART Goal #1 Plan:

Prior Active SMART Goal #1: ___________

Prior Active SMART Goal Domain #1: ___________

*Max of 2 active SMART goals at a time and within the same domain

- If the participant is creating a NEW goal they must be abandoning or retiring a prior goal or have no NEW or prior active goal selected. Please select ONLY one of these options.
- If the participant does not have a prior goal, you may not select continue, modify, abandon, or retire.
- If the participant is retiring a prior goal, you may not select continue, modify, or abandon.
- If the participant is abandoning a prior goal, you may not select continue or modify.

☐ No prior active goal #1
☐ Continue with prior active goal #1
☐ Modify (MINOR) prior active goal #1
☐ Abandon prior active goal #1
☐ Retire prior active goal #1 from sessions with M2H coach because goal is achieved!
☐ Retire prior active goal #1 from sessions with M2H coach because participant is now independently addressing the goal.
☐ Create a NEW Active SMART Goal #1
☐ Other

Explain "Other":

On a scale of 0 to 10, how well does the participant think their Prior Active SMART Goal #1 is going?

○ 0 (not well) ○ 1 ○ 2 ○ 3 ○ 4 ○ 5 ○ 6 ○ 7 ○ 8 ○ 9 ○ 10 (very well)

What barriers did the participant deal with since the last M2H session?

☐ None
☐ Lack of Time/Time Management Difficulty
☐ Unable to find/access resources
☐ Disinterested
☐ Overwhelmed
☐ Other

List the "Military Resource(s)":

List the "Community Resource(s)":

Explain "Other":

Prior Actions and Plans: ___________

Appointments since the last M2H Session. Select a box to represent each appointment:

i.e. If there are 3 visits, select 1 AND 2 AND 3

☐ 0
☐ 1
☐ 2
☐ 3
☐ 4
☐ 5
☐ 6
☐ 7
☐ 8
☐ 9

Prior Active SMART Goal #1: Previous Appointment

Clinic: Sleep, Nutrition, AWC, Chaplain, etc.

Type: 1:1, Class, etc.

Example: Sleep Clinic; 1:1

____
(Appointment Clinic; Type)

Days from Enrollment: _________

M2H Coach Follow-up

Active SMART Goal #1

Today's Active SMART Goal #1:

1. If continuing prior goal #1, copy/paste Prior Active SMART Goal as Active SMART Goal.

2. If modifying prior goal #1, copy/paste Prior Active SMART Goal and modify (minor modifications only).

3. If abandoning or retiring goal #1, ignore Prior Active SMART Goal and write in NEW Active SMART Goal #1.

Prior Active SMART Goal #1: *XXXXX*

____
(Today's Active SMART Goal)

If the patient has selected a NEW Active SMART Goal #1, please indicate the DOMAIN:

- ◯ Sleep
- ◯ Activity
- ◯ Nutrition
- ◯ Intrinsic
- ◯ Extrinsic

Actions and Plans for Active SMART Goal #1?

- ☐ Emailed participant new domain information
- ☐ Submitted EMR consult/referral
- ☐ Submitted M2H referral (non-EMR)
- ☐ Scheduled M2H follow-up session
- ☐ Introduced a military resource (i.e. class, website, app, phone number, literature)
- ☐ Introduced a community resource (i.e. class, website, app, phone number, literature)
- ☐ Continued with previous action/plan
- ☐ Other
- ☐ None

List the "Military Resource(s)":

List the "Community Resource(s)":

Explain "Other":

What type of barrier(s) and solution(s) to the given barrier(s) are anticipated by the participant for Active SMART Goal #1?

____

SMART Goal #2

SMART Goal #2 Plan:

Prior Active SMART Goal #2: N/A

Prior Active SMART Goal Domain #2: N/A

*Max of 2 active SMART goals at a time and within the same domain.

- ☐ No prior active goal #2
- ☐ Create a NEW Active SMART Goal #2
- ☐ Other

If the participant is creating a NEW goal they must have no prior active goal selected.

Explain "Other":

M2H Coach Follow-up

Active SMART Goal #2

Today's Active SMART Goal #2:

1. If continuing prior goal #2, copy/paste Prior Active SMART Goal as Active SMART Goal.

2. If modifying prior goal #2, copy/paste Prior Active SMART Goal and modify (minor modifications only).

3. If abandoning or retiring goal #2, ignore Prior Active SMART Goal and write in NEW Active SMART Goal.

Prior Active SMART Goal #2: N/A

____
(Today's Active SMART Goal)

If the patient has selected a NEW Active SMART Goal #2, please indicate the DOMAIN:

- ◯ Sleep
- ◯ Activity
- ◯ Nutrition
- ◯ Intrinsic
- ◯ Extrinsic

Actions and Plans for Active SMART Goal #2?

- ☐ Emailed participant new domain information
- ☐ Submitted EMR consult/referral
- ☐ Submitted M2H referral (non-EMR)
- ☐ Scheduled M2H follow-up session
- ☐ Introduced a military resource (i.e. class, website, app, phone number, literature)
- ☐ Introduced a community resource (i.e. class, website, app, phone number, literature)
- ☐ Continued with previous action/plan
- ☐ Other
- ☐ None

List the "Military Resource(s)":

List the "Community Resource(s)":

Explain "Other":

What type of barrier(s) and solution(s) to the given barrier(s) are anticipated by the participant for Active SMART Goal #2?

Consults / Referrals / Appointments

Who is the first consult/referral/appointment with?

____

This consult is associated with which DOMAIN?

- ☐ Sleep
- ☐ Activity
- ☐ Nutrition
- ☐ Intrinsic
- ☐ Extrinsic

Who is the second consult/referral/appointment with?

____

This consult is associated with which DOMAIN?

- ☐ Sleep
- ☐ Activity
- ☐ Nutrition
- ☐ Intrinsic
- ☐ Extrinsic

M2H Coach Follow-up

You are seeing a GROC for each domain the participant has ever set a SMART goal for. Please complete ALL GROCs that are visible.

With respect to __________ health how does the participant describe their current status since beginning Move to Health?

*This is NOT asking about their low back symptoms.

- ○ +7 A very great deal better
- ○ +6 A great deal better
- ○ +5 Quite a bit better
- ○ +4 Moderately better
- ○ +3 Somewhat better
- ○ +2 A little bit better
- ○ +1 A tiny bit better (almost the same)
- ○ 0 About the same
- ○ -1 A tiny bit worse (almost the same)
- ○ -2 A little bit worse
- ○ -3 Somewhat worse
- ○ -4 Moderately worse
- ○ -5 Quite a bit worse
- ○ -6 A great deal worse
- ○ -7 A very great deal worse

Coach

Follow-up Type:

- ○ In-person
- ○ Voice Only
- ○ Video
- ○ Other

Explain "Other":

Follow-up Duration (Minutes): __________

As the M2H coach, how would you gauge your participant's % investment in this intervention?

0% ———————————— 100%

(Place a mark on the scale above)

Coach Notes:

*Write any reminders here that you want to reference in the future follow up form!
*What challenges did you have?
*Participant's % confidence in achieving goal?
*What worked well for you?
*Details to remember about this participant; information for future interactions to help engage:
*Tools that proved to be effective today:
*Other:

Motivational Interviewing Self-Assessment: To what degree did you implement these components during your interaction today?

	0 Not at all	1	2	3	4	5	6	7	8	9	10 Grand Slam
Open Ended Questions	○	○	○	○	○	○	○	○	○	○	○
Affirmations	○	○	○	○	○	○	○	○	○	○	○
Reflective Listening (Change Talk)	○	○	○	○	○	○	○	○	○	○	○
Summaries	○	○	○	○	○	○	○	○	○	○	○
Suppressing Righting Reflex	○	○	○	○	○	○	○	○	○	○	○
Engage	○	○	○	○	○	○	○	○	○	○	○
Focus	○	○	○	○	○	○	○	○	○	○	○
Evoke	○	○	○	○	○	○	○	○	○	○	○
Plan	○	○	○	○	○	○	○	○	○	○	○

WHAT IS YOUR S.M.A.R.T. GOAL?

Empowering you to take control of your health and well-being

**S**	**SPECIFIC**	POINT OUT WITH CLARITY AND DETAIL EXACTLY WHAT IT IS THAT YOU WOULD LIKE TO ACHIEVE.
**M**	**MEASURABLE**	THE CRITERIA AND TOOLS YOU WILL USE TO MEASURE YOUR PROGRESS TOWARD ATTAINING YOUR GOAL.
**A**	**ATTAINABLE**	SET A GOAL THAT YOU HAVE THE SKILLS AND ABILITIES TO ACHIEVE BASED ON WHERE YOU ARE RIGHT NOW.
**R**	**REALISTIC**	HONESTLY CONSIDER YOUR TIME, MONEY, RESOURCES AND LEVEL OF SKILL TO CHOOSE A REALISTIC GOAL.
**T**	**TIME-BOUND**	SET A DEADLINE FOR THE ACHIEVEMENT OF YOUR GOAL AND OBJECTIVE USING A REASONABLE TIMELINE.

4 TYPES OF MOTIVATION

WHAT IS MOTIVATING YOU TO CHANGE?

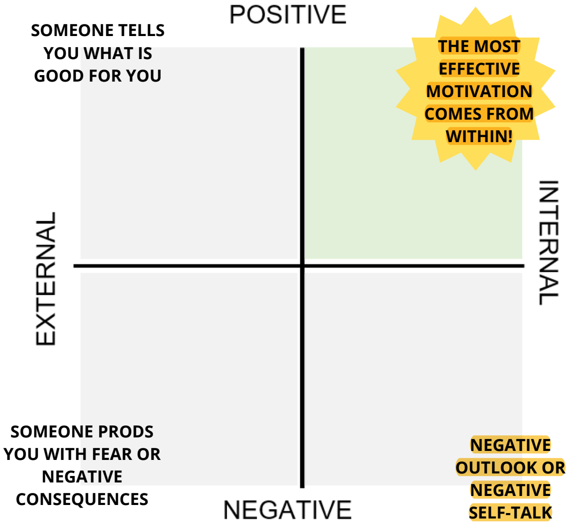

MY SMART GOAL FOR THE NEXT WEEK:

Things that might get in my way. Examples: weather, pain, time:

My strengths and resources I can use when my confidence is low:

MY MOVE TO HEALTH PLAN

- ☐ I will receive phone call check ins from my M2H Coach, ________________________.
- ☐ *Health Coach will write any pertinent information regarding referrals, etc. in this space.*
- ☐
- ☐ I have an in-person follow up with my M2H Coach, on ______________________ at _________________.

Research Contact Phone:

I BELIEVE THAT I CAN REACH MY GOAL

(Circle the number that matches how confident you feel)

1	2	3	4	5	6	7	8	9	10
Not at all				Somewhat sure					Very sure

Legal Disclaimer: The appearance of hyperlinks and downloadable applications does not constitute endorsement by the U.S. Army, of non-U.S. Government sites or the information products, or services contained therein. Although the U.S. Army may link to this site for informational purposes it does not exercise editorial control over the information that you may find on this site. Such links are provided consistent with the stated purpose of this document.

MOVE 2 HEALTH

A Holistic Approach to Managing Chronic Low Back Pain

Health is physical, mental and social well being. It is not the absence of disease or infirmity.

THE MOVE TO HEALTH APPROACH

SUPPORTS YOUR ABILITY TO CHOOSE WHAT ASPECTS OF YOUR HEALTH ARE IMPORTANT TO YOU. YOUR HEALTHCARE TEAM AIMS TO PARTNER WITH YOU TO HELP YOU ACHIEVE YOUR BEST HEALTH.

DID YOU KNOW?

YOUR ENVIRONMENT CONTRIBUTES TO ~20% OF YOUR OVERALL HEALTH.

WHAT IS HOLISTIC HEALTH?

A BALANCED INTERACTION OF ALL OF THESE DOMAINS. A HOLISTIC HEALTH PERSPECTIVE ENCOURAGES YOU TO TAKE CONTROL OF THE FUNCTIONS AND BEHAVIORS THAT AFFECT YOUR LIFE.

ACTIVITY

- **INACTIVITY IS COMMON IN INDIVIDUALS WITH CHRONIC LOW BACK PAIN AND ASSOCIATED WITH POORER RECOVERY.**
- **PHYSICAL ACTIVITY INTERVENTIONS FOR CHRONIC LOW BACK PAIN ARE SAFE AND EFFECTIVE.**
- **ADULTS WHO DO ANY AMOUNT OF MODERATE TO VIGOROUS ACTIVITY GAIN HEALTH BENEFITS.**

HOW MUCH DO YOU MOVE DURING THE DAY?

SLEEP

- **MANY PEOPLE, PARTICULARLY ACTIVE DUTY, DO NOT GET ADEQUATE SLEEP.**
- **SLEEP DYSFUNCTION IS ASSOCIATED WITH CHRONIC LOW BACK PAIN.**
- **EVIDENCE SUGGESTS ADDRESSING SLEEP DYSFUNCTION MAY FACILITATE LOW BACK PAIN RECOVERY.**

ARE YOU GETTING 7-8 HOURS/NIGHT?

NUTRITION

- **POOR DIETS NEGATIVELY IMPACT LOW BACK PAIN AS A RESULT OF INFLAMMATORY EFFECTS, VITAMIN DEFICIENCIES AND OVER-RELIANCE ON CAFFEINE.**
- **POOR NUTRITIONAL HABITS CAN LEAD TO OBESITY - A RISK FACTOR FOR LOW BACK PAIN.**

DO YOU EAT NUTRIENT-RICH FOODS LIKE FRUITS AND VEGETABLES REGULARLY?

FAMILY & SOCIAL LIFE

- **RELATIONSHIPS CAN INFLUENCE YOUR DECISION MAKING – IN GROUP SETTINGS YOU ARE MORE LIKELY TO COPY THE BEHAVIORS OF THOSE AROUND YOU.**
- **SOCIAL SUPPORT INCREASES THE LIKELIHOOD OF RECOVERING FROM LOW BACK PAIN.**

HOW IS THE QUALITY/HEALTH OF YOUR CURRENT RELATIONSHIPS?

PERSONAL DEVELOPMENT

- **STRESS AND LOW MOOD ASSOCIATED WITH WORK/LIFE DISSATISFACTION CAN CONTRIBUTE TO LOWER OVERALL HEALTH AND CHRONIC PAIN.**

ARE YOU FEELING FULFILLED IN YOUR WORK/PERSONAL LIFE?

- **PEOPLE WITH LOW BACK PAIN WHO QUIT SMOKING DURING CARE HAD GREATER PAIN RELIEF COMPARED TO THOSE WHO KEPT SMOKING.**

DO YOU WANT HELP QUITTING TOBACCO?

SPIRITUAL

- **ANGER, DESPAIR, SADNESS, ANXIETY AND DEPRESSION MAY BE SIGNS OF SPIRITUAL DISTRESS.**

WHERE DO YOU FIND PURPOSE, BELONGING AND SUPPORT DURING HARD TIMES?

EMOTIONAL

- **STRESS, DISTRESS, DEPRESSION AND ANXIETY ARE KNOWN RISK FACTORS FOR CHRONIC LOW BACK PAIN.**
- **STRESS REDUCTION, LEARNING NEW COPING SKILLS AND MONITORING UNHELPFUL THOUGHTS/FEELINGS CAN HELP IMPROVE LOW BACK PAIN.**

HOW WELL DO YOU REGULATE YOUR EMOTIONS?

SURROUNDINGS

- **ENVIRONMENTS IMPACT MOOD, BEHAVIOR, STRESS, SLEEP, AND HEALTH**
- **BEING OUTSIDE IN THE SUN AND FRESH AIR CAN IMPROVE PAIN LEVELS.**

DO YOUR HOME, WORK, PERSONAL AND SOCIAL COMMUNITY FEEL SAFE, CLEAN, COMFORTABLE, ACCESSIBLE AND FUNCTIONAL?

Legal Disclaimer: The appearance of hyperlinks and downloadable applications does not constitute endorsement by the U.S. Army, of non-U.S. Government sites or the information products, or services contained therein. Although the U.S. Army may link to this site for informational purposes it does not exercise editorial control over the information that you may find on this site. Such links are provided consistent with the stated purpose of this document.

3 QUESTIONS FOR CHANGE!

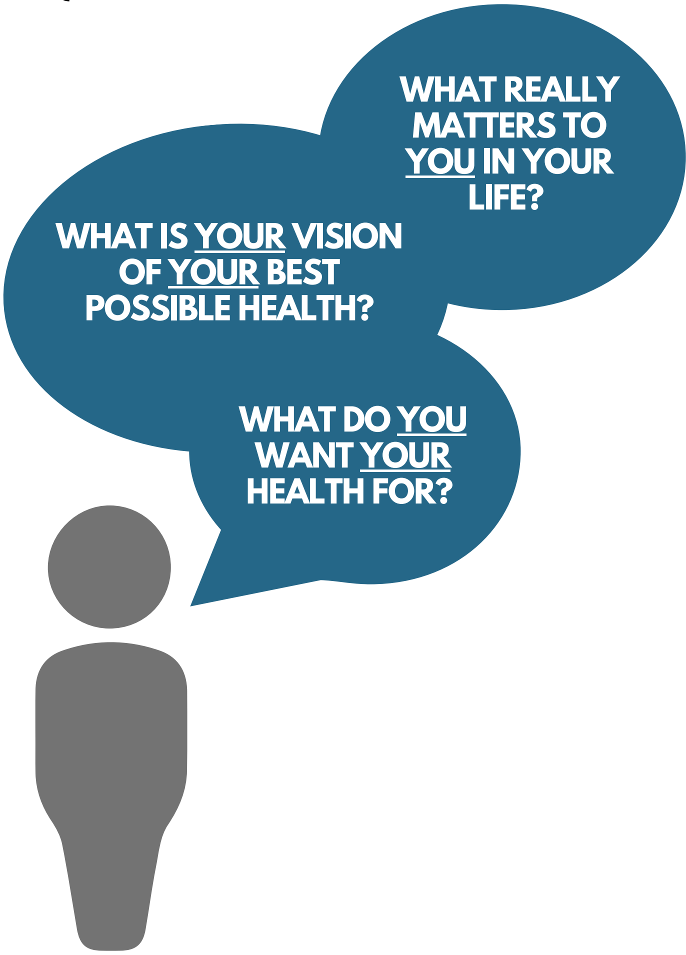

ACTIVITY

EXERCISE GIVES YOU ENERGY AND STRENGTH. MOVEMENT CAN MAKE YOU MORE FLEXIBLE. EXERCISE IS ALSO GOOD FOR YOUR MIND. REGULAR EXERCISE CAN LOWER BLOOD PRESSURE, CHOLESTEROL AND REDUCE THE RISK FOR HEART DISEASE. EXAMPLES OF EXERCISE AND MOVEMENT INCLUDE WALKING, GARDENING, DANCING, OR LIFTING WEIGHTS. FIND WHAT WORKS FOR YOU.

"ENERGY & FLEXIBILITY"

NUTRITION

WHAT YOU EAT AND DRINK NOURISHES YOUR BODY AND MIND. CHOOSE HEALTHY EATING HABITS THAT FIT YOUR LIFESTYLE. LIMIT ALCOHOL, CAFFEINE, AND NICOTINE. KEEP YOUR BODY AND MIND PROPERLY FUELED. LIMIT ADDED SUGARS IN YOUR DIET.

"NOURISHING & FUELING"

SLEEP

SLEEP IS VERY IMPORTANT FOR YOUR BODY AND MIND. REST CAN GIVE YOU PEACE. RELAXATION CAN LOWER STRESS. ACTIVITIES YOU ENJOY CAN HELP YOU FEEL RECHARGED. A GOOD BALANCE BETWEEN ACTIVITY AND REST IMPROVES YOUR HEALTH AND WELL-BEING.

"REST & RECHARGE"

PERSONAL DEVELOPMENT

NO MATTER WHERE YOU ARE IN LIFE, YOUR PERSONAL & WORK LIFE ARE VERY IMPORTANT. HOW DO YOU SPEND YOUR TIME & ENERGY DURING THE DAY? DO THINGS GIVE YOU ENERGY OR MAKE YOU TIRED? DO YOU SPEND TIME DOING WHAT MATTERS MOST TO YOU? HOW DO YOU FEEL ABOUT YOUR FINANCES AND HOW ARE THEY AFFECTING YOUR LIFE? THESE FACTORS AFFECT NOT ONLY YOUR HAPPINESS, BUT ALSO YOUR HEALTH.

"PERSONAL LIFE & WORK LIFE"

FAMILY & SOCIAL LIFE

FEELING ALONE CAN SOMETIMES MAKE YOU GET SICK OR KEEP YOU SICK. POSITIVE SOCIAL RELATIONSHIPS ARE HEALTHY. A HEALTHY INTIMATE RELATIONSHIP WITH A LIFE PARTNER CAN BE A SOURCE OF STRENGTH. IT'S GOOD TO TALK TO PEOPLE WHO CARE ABOUT YOU AND LISTEN TO YOU.

"RELATIONSHIPS"

"ONLY I CAN KNOW WHY I WANT MY HEALTH. ONLY I CAN KNOW WHAT REALLY MATTERS TO ME.
THIS KNOWLEDGE NEEDS TO BE WHAT DRIVES MY HEALTH AND MY HEALTHCARE. I AM THE MOST IMPORTANT PERSON WHEN IT COMES TO MAKING CHOICES THAT INFLUENCE MY HEALTH AND WELL-BEING."

SPIRITUAL

A SENSE OF MEANING AND PURPOSE IN LIFE IS IMPORTANT TO MANY PEOPLE. WHEN THINGS ARE HARD, WHERE DO YOU TURN FOR STRENGTH AND COMFORT? SOME PEOPLE TURN TO SPIRITUAL OR RELIGIOUS FAITH. SOME PEOPLE FIND COMFORT IN NATURE. SOME CONNECT WITH ART, MUSIC OR PREFER QUIET TIME ALONE. SOME WANT TO HELP OTHERS. YOU MAY EXPRESS THIS AS A GUIDE TO LIVING FULLY.

"GROWING & CONNECTING"

EMOTIONAL

YOUR MIND CAN AFFECT YOUR BODY. SOMETIMES WHEN YOU THINK ABOUT STRESSFUL THINGS, YOUR HEART RATE AND BLOOD PRESSURE GO UP. YOU CAN USE THE POWER OF YOUR MIND TO LOWER BLOOD PRESSURE OR CONTROL PAIN. LEARN TO USE THE CONNECTION BETWEEN YOUR MIND AND BODY. WARRIORS AND ATHLETES USE THE POWER OF THE MIND TO VISUALIZE A SUCCESSFUL MISSION OR EVENT. MIND-BODY PRACTICES TAP INTO THE POWER OF THE MIND TO HEAL AND COPE.

"POWER OF THE MIND

SURROUNDINGS

YOUR ENVIRONMENT CAN AFFECT YOUR HEALTH. YOU MAY HAVE PROBLEMS WITH SAFETY, OR THINGS LIKE CLUTTER, NOISE BAD SMELLS, POOR LIGHTING OR WATER QUALITY. YOU MAY BE ABLE TO CHANGE SOME OF THESE PROBLEMS. YOU MAY NOT BE ABLE TO CHANGE THEM ALL. IT STARTS WITH PAYING ATTENTION TO THE INFLUENCES OF YOUR ENVIRONMENT ON YOUR LIFE AND HEALTH. IMPROVE WHAT YOU CAN. IT'S GOOD TO HAVE A SAFE, COMFORTABLE, AND HEALTHY SPACE.

"PHYSICAL & EMOTIONAL"

Legal Disclaimer: The appearance of hyperlinks and downloadable applications does not constitute endorsement by the U.S. Army, of non-U.S. Government sites or the information products, or services contained therein. Although the U.S. Army may link to this site for informational purposes it does not exercise editorial control over the information that you may find on this site. Such links are provided consistent with the stated purpose of this document.

MOVE 2 HEALTH

A GUIDE TO WALKING FOR LOW BACK PAIN

Keeping an Active Lifestyle

TARGETS FOR OPTIMAL HEALTH

10,000 — **Steps per day**
(1 mile = ~2,000 steps)
The average American step count is ~4,000/day

<4 hrs — **Sit LESS than 4 hours per day**

150 min — **Of moderate aerobic activity a week is equal to a brisk walk at an effort which allows you to talk but not sing**

DO THESE TARGETS SEEM TOO DIFFICULT?

That is OK! Small, incremental increases in activity can still have **BIG BENEFITS!**

Walking can help **lower stress, increase energy, relieve pain, boost your mood, and improve heart health.**

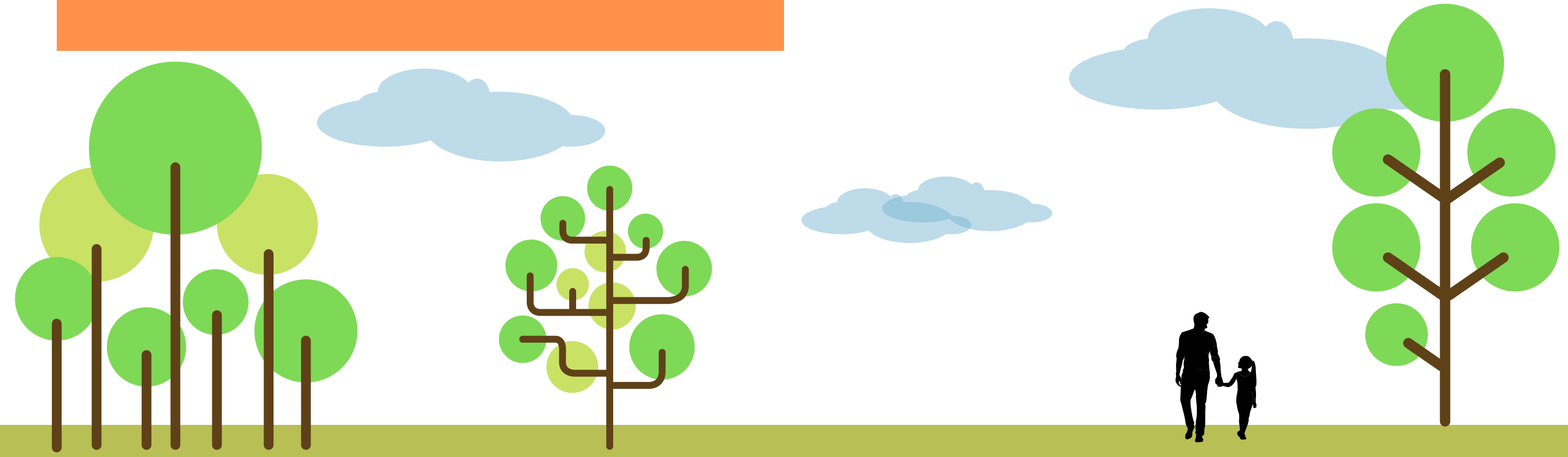

GO GREEN!

Research suggests that walking in natural green spaces can provide EXTRA psychological benefits!

Legal Disclaimer: The appearance of hyperlinks and downloadable applications does not constitute endorsement by the U.S. Army, of non-U.S. Government sites or the information products, or services contained therein. Although the U.S. Army may link to this site for informational purposes it does not exercise editorial control over the information that you may find on this site. Such links are provided consistent with the stated purpose of this document.

HOW DO I GET STARTED?

1. GET A CHECK UP

Consult with your medical provider if you have any concerns about high blood pressure, diabetes, heart conditions, chest pain, and/or dizziness with exertion.

2. SET SMART GOALS

S - SPECIFIC
M - MEASURABLE
A - ATTAINABLE
R - REALISTIC
T - TIMELY

"I will walk to the end of my street and back before I bring in the mail every day this week."

3. MAKE A PLAN

- Organize your day to incorporate walking
- Invite others to join you
- Locate safe and convenient places to walk
- Wear proper footwear and comfortable clothing

4. WALK!

Have fun, track your progress, and celebrate your healthy choices!

RESEARCH SHOWS

Walking helps improve pain, function, and quality of life in patients with chronic low back pain with a very low risk of injury.

ADDITIONAL RESOURCES

SAMPLE OF AN 8 WEEK WALKING PROGRAM
HTTPS://WWW.NHLBI.NIH.GOV/HEALTH/EDUCATIONAL/HEALTHDISP/PDF/TIPSHEETS/SAMPLE-WALKING-PROGRAM.PDF

INFO ABOUT HEALTH BENEFITS OF WALKING, NEWS, VIDEOS, & EVENTS
EVERYBODYWALK.ORG

Legal Disclaimer: The appearance of hyperlinks and downloadable applications does not constitute endorsement by the U.S. Army, of non-U.S. Government sites or the information products, or services contained therein. Although the U.S. Army may link to this site for informational purposes it does not exercise editorial control over the information that you may find on this site. Such links are provided consistent with the stated purpose of this document.

MOVE 2 HEALTH

HOW TO IMPROVE YOUR SLEEP

Sleep Positioning and Considerations for Low Back Pain

THE WAYS SLEEP IMPACTS YOU

Lack of sleep is linked to UNHEALTHY FOOD CHOICES and increases the risk of OBESITY by 21%

Proper sleep OPTIMIZES your cognitive and physical PERFORMANCE

Proper sleep can decrease the risks of CORONARY HEART DISEASE

Lack of sleep slows down injury recovery and INCREASES LEVELS OF PAIN

Losing 4 OR MORE hours of sleep is the equivalent of DRIVING DRUNK

Proper sleep DECREASES chronic pain, high BP, diabetes, stroke, and other CHRONIC DISEASES

With proper sleep you will have a STRONGER immune system

Lack of sleep makes it harder to interpret emotions and relate with others, putting a STRAIN ON RELATIONSHIPS

HOW MANY HOURS OF SLEEP DO I NEED?

TEENAGERS
8-10
14-17 YRS

ADULTS
7-9
18-64 YRS

OLDER ADULT
7-8
65+ YRS

DOES MY MATTRESS MATTER?

Patients report improved low back pain and a better night's sleep when they sleep on a mattress described as **MEDIUM-FIRM**

Replace your mattress every **10 YEARS**

Replace your pillows every **2 YEARS**

Buying an expensive mattress is not necessary.

Americans who are very motivated to get enough sleep reported sleeping 36 MORE MINUTES per night across the week!

Legal Disclaimer: The appearance of hyperlinks and downloadable applications does not constitute endorsement by the U.S. Army, of non-U.S. Government sites or the information products, or services contained therein. Although the U.S. Army may link to this site for informational purposes it does not exercise editorial control over the information that you may find on this site. Such links are provided consistent with the stated purpose of this document.

A PROPER SLEEP POSITION MAINTAINS ALIGNMENT OF EARS, SHOULDERS AND HIPS WITH LITTLE ROTATION OF THE SPINE

ESTABLISH A WAKE AND SLEEP SCHEDULE AND STICK TO IT EVEN ON **WEEKENDS**

STRIVE TO GET UP AND MOVE AROUND DURING THE DAY; A STIFF BACK MAY INCREASE DISCOMFORT AT BEDTIME

UNWIND **30 MIN** BEFORE BED BY PUTTING AWAY ELECTRONICS

Lying on your BACK

Allows for neutral spine and even weight distribution. A pillow under the neck and knees may add additional comfort

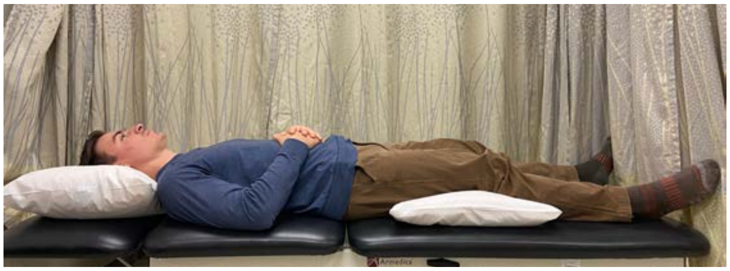

Lying on your SIDE

This is the most common sleep position. A pillow between the knees or under the shoulder will help prevent the spine from bending or rotating

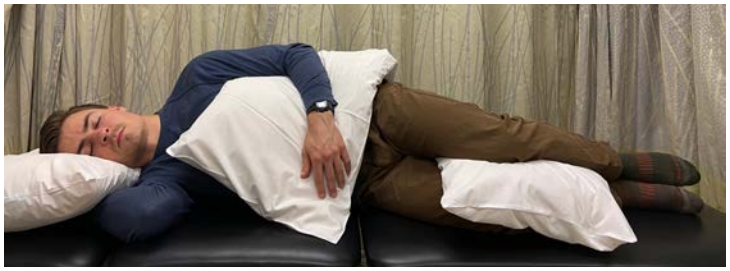

Lying on your STOMACH

This position is generally NOT recommended. A pillow under the abdomen and/or lower legs may help decrease stress on the low back.

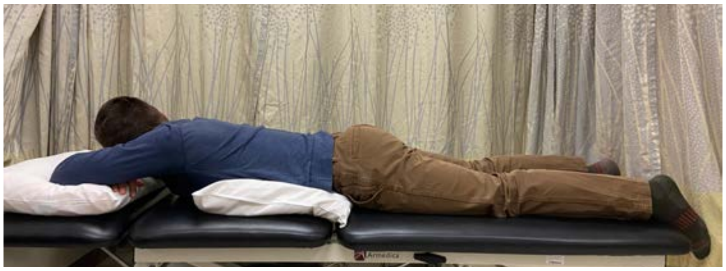

**Note: Sleep position alone neither causes nor alleviates low back pain.

ADDITIONAL RESOURCES

NIH

YOUR GUIDE TO HEALTHY SLEEP FROM THE NIH

HTTPS://WWW.NHLBI.NIH.GOV/FILES/DOCS/PUBLIC/SLEEP/HEALTHYSLEEPFS.PDF

Sleep.org BY THE NATIONAL SLEEP FOUNDATION

PROVIDES INFORMATION VIA GRAPHICS AND ARTICLES ABOUT SLEEP AND HOW TO HELP YOURSELF GET THE RIGHT AMOUNT OF SLEEP.

Legal Disclaimer: The appearance of hyperlinks and downloadable applications does not constitute endorsement by the U.S. Army, of non-U.S. Government sites or the information products, or services contained therein. Although the U.S. Army may link to this site for informational purposes it does not exercise editorial control over the information that you may find on this site. Such links are provided consistent with the stated purpose of this document.

MOVE 2 HEALTH

DEEP BREATHING EXERCISES

For Relaxation and Pain Relief

Research shows that practicing deep breathing has many restorative health benefits such as:

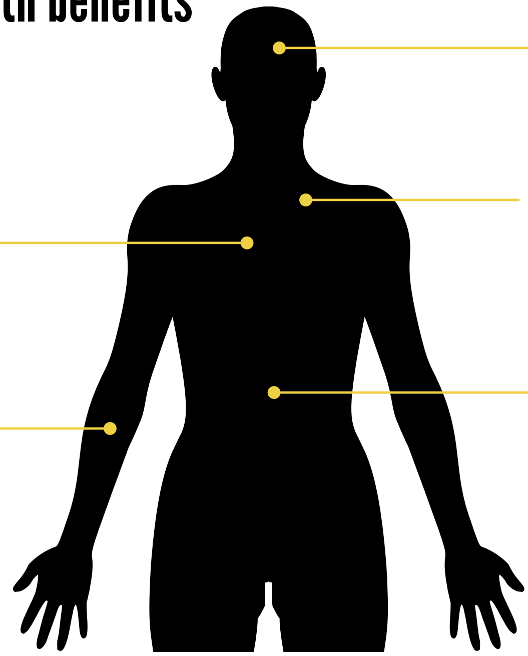

DID YOU KNOW?

- People with chronic low back pain may also demonstrate inefficient and abnormal breathing patterns
- Deep breathing helps sharpen focus and memory for a productive day
- Deep breathing, pain, heart/lung function and emotions are closely linked
- Deep breathing decreases levels of stress hormones for a stronger immune system
- Deep breathing promotes positive thoughts, calmness, alertness, and decreases stress and anger

"Remember to breathe. It is after all, the secret of life." - Author Gregory Maguire

Legal Disclaimer: The appearance of hyperlinks and downloadable applications does not constitute endorsement by the U.S. Army, of non-U.S. Government sites or the information products, or services contained therein. Although the U.S. Army may link to this site for informational purposes it does not exercise editorial control over the information that you may find on this site. Such links are provided consistent with the stated purpose of this document.

HOW DO I GET STARTED?

1 Start in a **comfortable** position, which can be lying down or sitting.

2 **Slowly inhale** through your nose using your **diaphragm** (rather than your chest) to draw in air. Try to **feel your stomach rise** without feeling your chest move.

3 **Slowly exhale** through your mouth. Allow your stomach to return to start **without moving your chest**.

4 **Repeat** the cycle as many times as you need to **feel relaxed**. Concentrate on the sensation of your breath and **remove outside distractions.**

Slow your breathing rate to about 6 to 10 breaths per minute

RELAX

Deep breathing is most effective when performed in a relaxed state

- FIND A QUIET SPACE
- TRY YOGA
- USE CALMING SCENTS
- TAKE A WARM BATH
- SIT OR TAKE A WALK IN THE PARK

ADDITIONAL RESOURCES

VA U.S. Department of Veterans Affairs

GET IT ON Google Play

Available on the iPhone App Store

MORE ON BREATHING AND HEALTH FROM THE VA

HTTPS://WWW.VA.GOV/PATIENTCENTEREDCARE/ VETERAN-HANDOUTS/BREATHING_AND_HEALTH.ASP

Breath2Relax App

TEACHES BREATHING TECHNIQUES AS A TOOL FOR STRESS REDUCTION AVAILABLE FOR IOS AND ANDROID

Legal Disclaimer: The appearance of hyperlinks and downloadable applications does not constitute endorsement by the U.S. Army, of non-U.S. Government sites or the information products, or services contained therein. Although the U.S. Army may link to this site for informational purposes it does not exercise editorial control over the information that you may find on this site. Such links are provided consistent with the stated purpose of this document.

RESOURCES TO AIDE YOUR
ACTIVE LIFE STYLE

Empowering you to take control of your health and well-being

GENERAL ONLINE RESOURCE

Move Your Way

Find tools, videos, and fact sheets to help you make small changes in your activity level to reap big health benefits!

MOVE YOUR WAY
health.gov

MILITARY-SPECIFIC RESOURCES

MILITARY ONESOURCE
www.militaryonesource.mil/health-wellness

A website with fresh, practical content for developing strategies to live an active lifestyle and stay fit.

HPRC HUMAN PERFORMANCE RESOURCE CENTER
www.hprc-online.org/page/physical-fitness

Articles and resources about physical conditioning to maintain performance, prevent injury and illness, and speed recovery.

Performance Triad (P3) App

Learn how to change your daily sleep, ACTIVITY and nutrition routines to become stronger, faster, leaner and mentally sharper.

GET IT ON Google Play
Available on the iPhone App Store

Get the PDF of the P3 Challenge Book Here!

BAMC/FSH RESOURCES

MWR-Recreation Facility

https://www.jbsatoday.com/RecreationTravel

Golf Course
Bowling Center
Equestrian Center
Outdoor Recreation Center

Sports, Fitness, & Aquatics Facility

https://www.jbsatoday.com/FSH-Fitness

Aquatic Center
Fitness Center on the METC
Jimmy Brought Fitness Center
Central Post Fitness Center

Armed Forces Wellness Center

Call to Schedule: (210) 539-1254
Location: 2490 Stanley Rd, San Antonio, TX 78234
(Inside the Vogel Resiliency Center)

Staying Fit Home & Away Class
BodPod Health Assessment
SubMax VO2 Fitness Assessment
Individualized Exercise Plan
Exercise Counseling

WRITE IT DOWN!
WHY DO YOU WANT TO MAKE ACTIVITY A PRIORITY?

Remember, EVERYONE benefits from physical activity including those with pain. Start low and go slow. Even 5 minutes of daily activity has REAL health benefits!

For Aerobic Activities:
Aim for at least 150 min/week of moderate intensity OR 75 min/week of vigorous intensity.

For Strength Training:
Aim for strengthening activities of all major muscle groups 2 times/week

Legal Disclaimer: The appearance of hyperlinks and downloadable applications does not constitute endorsement by the U.S. Army, of non-U.S. Government sites or the information products, or services contained therein. Although the U.S. Army may link to this site for informational purposes it does not exercise editorial control over the information that you may find on this site. Such links are provided consistent with the stated purpose of this document.

RESOURCES TO HELP
IMPROVE YOUR SLEEP

Empowering you to take control of your health and well-being

ONLINE RESOURCE & SLEEPING AID

Sleep.org BY THE NATIONAL SLEEP FOUNDATION

Sleep.org is a website with easy to understand graphics explaining:

- The science behind why our bodies need sleep
- How to design a bedroom that promotes sleep
- Lifestyle behaviors that help and hurt sleep

COGNITIVE BEHAVIORAL THERAPY FOR INSOMNIA

Veterans Affairs Path to Better Sleep**

veterantraining.va.gov/insomnia/index.asp

Free interactive website to help address insomnia. It also includes:

Path to Better Sleep

- Information on sleep
- Tips to develop good sleep habits and modify behaviors that interfere with sleep
- Testimonial videos from veterans and much more information about insomnia

** May be used on its own, but not intended to replace therapy.

MILITARY-SPECIFIC RESOURCES

Breathe2Relax App

Provides interactive instructions on how to use breathing to promote relaxation. Perfect for unwinding before sleep.

Performance Triad App

Learn how to change your daily SLEEP, activity and nutrition routines to become stronger, faster, leaner and mentally sharper.

GET IT ON Google Play

Available on the iPhone App Store

Performance Triad Challenge Book!

BAMC RESOURCES

Armed Forces Wellness Center

Call to Schedule: (210) 539-1254

Location: 2490 Stanley Rd, San Antonio, TX 78234

(Inside the Vogel Resiliency Center)

Healthy Sleep Habit Classes

- Held twice a month
- Explore the science of sleep
- Methods for better quality sleep
- Create an action plan for improved sleep

WRITE IT DOWN!
WHY DO YOU WANT TO MAKE SLEEP A PRIORITY?

Legal Disclaimer: The appearance of hyperlinks and downloadable applications does not constitute endorsement by the U.S. Army, of non-U.S. Government sites or the information products, or services contained therein. Although the U.S. Army may link to this site for informational purposes it does not exercise editorial control over the information that you may find on this site. Such links are provided consistent with the stated purpose of this document.

RESOURCES TO HELP

FUEL YOUR PERFORMANCE & HEALTH

Empowering you to take control of your health and well-being

MILITARY SPECIFIC RESOURCES

MILITARY ONESOURCE
https://www.militaryonesource.mil/health-wellness/healthy-living

Provides practical and fresh content as well as initiatives and programs that will motivate you to maintain healthy living and manage your health in creative ways.

HPRC
HUMAN PERFORMANCE RESOURCE CENTER
https://www.hprc-online.org/nutrition/performance-nutrition

Learn about how nutrition basics and specialty topics enhance Warfighter performance during workouts, training, missions, and recovery.

Performance Triad (P3) App
Learn how to change your daily sleep, activity and NUTRITION. routines to become stronger, faster, leaner and mentally sharper.

Available on the iPhone App Store

GET IT ON Google Play

TRACKING TOOLS & APPLICATION

Choose MyPlate
Helps you find your healthy eating style that fits your individual needs.
Choosemyplate.gov

The VA Health's Move! Coach App
Offers a 19 week program with educational and tracking tools for weight, diet and activity goals.

DOWNLOADABLE GUIDES

Army Cooking Guide.pdf

Navy Cooking Guide.pdf

P3 Challenge Book.pdf

BAMC/FSH RESOURCES

Nutrition Clinic

Call to Make Appointment: (210) 808-2232

Self Refer for Individual Appointments & Recipe Ideas!

Armed Forces Wellness Center

Call to Schedule: (210) 539-1254
Location: 2490 Stanley Rd, San Antonio, TX 78234
(Inside the Vogel Resiliency Center)

Meals in Minutes Class
Fueling Your Health Class
Upping Your Metabolism Class

WRITE IT DOWN!
WHY DO YOU WANT TO MAKE YOUR NUTRITION A PRIORITY?

Legal Disclaimer: The appearance of hyperlinks and downloadable applications does not constitute endorsement by the U.S. Army, of non-U.S. Government sites or the information products, or services contained therein. Although the U.S. Army may link to this site for informational purposes it does not exercise editorial control over the information that you may find on this site. Such links are provided consistent with the stated purpose of this document.

MOVE 2 HEALTH

RESOURCES TO HELP
STRENGTHEN RELATIONSHIPS

Empowering you to take control of your health and well-being

MILITARY SPECIFIC RESOURCES

MILITARY ONESOURCE

https://www.militaryonesource.mil/family-relationships

A website with with confidential counseling and resources to support and bolster your family relationships.

REAL WARRIORS ★ REAL BATTLES REAL STRENGTH

https://www.realwarriors.net/family-relationship?page=1

The Real Warriors Campaign provides resources to learn how to develop and maintain strong relationships and keep your family healthy even while facing challenges.

Safe Helpline 877-995-5247

www.safehelpline.org

24/7 Sexual Assault Support for the DoD Community ANONYMOUS. CONFIDENTIAL.

BUILDING SOCIAL RELATIONSHIPS

happify DAILY

https://www.happify.com/hd/strengthen-your-relationships/

"Other People Matter" article for well-being and tips for strengthening relationships.

HPRC HUMAN PERFORMANCE RESOURCE CENTER

https://www.hprc-online.org/social-fitness/relationship-building

Articles and resources to support social fitness of Warfighter health and performance.

BAMC/FSH RESOURCES

Department of Behavioral Health

Call for group start dates: (210)916-1600

Walk-In Services are available. Known emergencies should go to the nearest Emergency Room

Military & Family Readiness Center

Call for More Information: (210)221-2705
3060 Stanley Road, Bldg 2797

-Parenting workshops
-Unit Family Readiness
-Support Groups for Single Parents & Special Needs Caregivers
-Adoption and Foster care seminars (LAK)

MWR-JBSA Events

https://jbsatoday.com/

- Social Events
- Family Friendly Events
- Family Advocacy Program
- Better Opportunities for Single Service Members (BOSS)
& much more!

CONTINUE ON BACK →

Legal Disclaimer: The appearance of hyperlinks and downloadable applications does not constitute endorsement by the U.S. Army, of non-U.S. Government sites or the information products, or services contained therein. Although the U.S. Army may link to this site for informational purposes it does not exercise editorial control over the information that you may find on this site. Such links are provided consistent with the stated purpose of this document.

RESOURCES FOR CHILDREN

Military Kids Connect

https://health.mil/MKCHome

Online community for military children ages 6-17 years old to access age-appropriate resources to cope with unique challenges of military life.

for Military Families

https://sesamestreetformilitaryfamilies.org/

Sesame Street for Military Families is a free, bilingual (English and Spanish) website where families can find information and multimedia resources on the topics of military deployments, multiple deployments, homecomings, injuries, grief, and self-expression.

WRITE IT DOWN!

WHY DO YOU WANT TO MAKE YOUR RELATIONSHIPS A PRIORITY?

PARENTING RESOURCES

Parenting2GO

(veterantraining.va.gov/parenting)

This training is also available as an app for iOS!

A free online course with tools to help service members and veterans strengthen their parenting skills and help them reconnect with their children.

INCLUDES 6 MODULES TO:

- Address everyday issues & issues unique to military life
- Promote positive parent-child communication
- Help children cope with difficult emotions & behaviors
- Education on a positive approach to discipline
- Manage parental stress and emotions
- Guide parenting when parents have emotional and physical challenges

Legal Disclaimer: The appearance of hyperlinks and downloadable applications does not constitute endorsement by the U.S. Army, of non-U.S. Government sites or the information products, or services contained therein. Although the U.S. Army may link to this site for informational purposes it does not exercise editorial control over the information that you may find on this site. Such links are provided consistent with the stated purpose of this document.

RESOURCES TO ASSIST WITH YOUR
PERSONAL DEVELOPMENT

Empowering you to take control of your health and well-being

MILITARY SPECIFIC RESOURCES

MILITARY ONESOURCE
https://www.militaryonesource.mil

Military OneSource Provides strategies for financial security and offers resources for pursuing education goals, achieving career aspirations, and improving leadership skills.

HPRC
HUMAN PERFORMANCE RESOURCE CENTER
https://www.hprc-online.org/social-fitness/teams-leadership

Find information to help you lead and work effectively with others on duty and in professional settings.

BAMC/FSH RESOURCES

Military & Family Readiness Center

Call for More Information: (210)221-2705
3060 Stanley Road, Bldg 2797

Resume writing
Career Fairs
Financial Planning
Relocation Planning
Volunteering Opportunities
Deployment Personal & Family Readiness

ONLINE VA COURSE

MOVING FORWARD
Overcoming Life's Challenges

MOVING FORWARD
A free online course for Veterans & Service Members that teaches skills to help overcome stressful problems and meet your goals.

Download the Moving Forward iOS app for problem solving on-the-go.

8 MODULES WITH VIDEOS, GAMES & INTERACTIVE EXERCISES:

Introduction to the program
Self-assessment for problem-solving
Stress Management
How to solve problems step-by-step
Steps to begin right away

A GREAT TOOL FOR FACING CHALLENGES LIKE:

Managing stress
Balancing work & family
Strained relationships
Physical injuries
Financial difficulties
Adjustment issues

WRITE IT DOWN!

WHY DO YOU WANT TO MAKE YOUR PERSONAL DEVELOPMENT A PRIORITY?

SELF-HELP PODCAST

BORNE THE BATTLE

Launched in late 2016 by the Department of Veterans Affairs. "Borne the Battle" recognizes each battle, challenge, and sacrifice our Veterans endure during and after their service, as well as spotlighting important resources, offices, and benefits VA offers our Veterans.

CONTINUE ON BACK

Legal Disclaimer: The appearance of hyperlinks and downloadable applications does not constitute endorsement by the U.S. Army, of non-U.S. Government sites or the information products, or services contained therein. Although the U.S. Army may link to this site for informational purposes it does not exercise editorial control over the information that you may find on this site. Such links are provided consistent with the stated purpose of this document.

RESOURCES FOR TOBACCO CESSATION

YOU CAN QUIT!

Empowering you to take control of your health and well-being

MILITARY SPECIFIC RESOURCES

YOU CAN QUIT2
https://www.ycq2.org/

You Can QUIT2's mission is to help service members quit tobacco-for themselves and their loved ones.
The site includes educational material, a 24/7 Live Chat with coaches, a text message program, and tips to help you quit and stay quit.

TRICARE
https://www.tricare.mil/TobaccoCessationServices

TRICARE provides its beneficiaries services, drugs and counseling to help them quit tobacco. Remember, when you combine counseling with cessation medication, you give yourself the best chance at quitting for good.

VA | U.S. Department of Veterans Affairs
1-855-QUIT-VET (1-855-784-8838)

VA's free quitline, **1-855-QUIT-VET**, offers tobacco cessation counseling to any Veteran who receives their health care through VA. Quit VET has trained counselors who will help during any phase of quitting. Quit VET counseling is offered in both English and Spanish.

SMARTPHONE APPLICATIONS

VA HEALTH'S STAY QUIT COACH APP
Provides information on smoking and quitting, interactive tools to help users cope with urges to smoke, motivational messages and support contacts to help you stay smoke-free.

Download on the App Store | GET IT ON Google Play

Download on the App Store | GET IT ON Google Play

QUITGUIDE APP FROM SMOKEFREE.GOV
Offers on the go access to the websites information. Features craving, slips, and trigger trackers, motivational messages, tips and guided help to identify reasons for quitting and staying quit.

DID YOU KNOW?

"Electronic Cigarettes, AKA Vaping, are just as toxic to your health as cigarettes and extremely addictive!"
Download to see what the **CDC** has to say about E-Cigarettes.

"Smokeless tobacco, AKA spit, chew and dip all contain the same harmful cancer causing ingredients as cigarettes! "
Download for a guide from **quitline.com** with the affects of smokeless tobacco and how to quit

BAMC/FSH RESOURCES

Vogel Resiliency Center

Call for more information: (210)539-1281
2490 Stanley Rd Bldg 367

Offers a Tobacco Cessation program designed to give you the tools you need for success.

WRITE IT DOWN!
WHY DO YOU WANT TO QUIT SMOKING OR USING TOBACCO?

Legal Disclaimer: The appearance of hyperlinks and downloadable applications does not constitute endorsement by the U.S. Army, of non-U.S. Government sites or the information products, or services contained therein. Although the U.S. Army may link to this site for informational purposes it does not exercise editorial control over the information that you may find on this site. Such links are provided consistent with the stated purpose of this document.

RESOURCES FOR ADDRESSING

EMOTIONAL & SPIRITUAL HEALTH

Empowering you to take control of your health and well-being

MILITARY RESOURCES

MILITARY ONESOURCE
https://www.militaryonesource.mil/confidential-help

For additional 24/7 access to confidential counseling and health coaching contact Military OneSource.

HPRC
HUMAN PERFORMANCE RESOURCE CENTER
https://www.hprc-online.org/mental-fitness/mental-health

Mental health is important to optimize your performance. Use these resources to help improve your mental health and grow from adversity.

REAL WARRIORS ★ REAL BATTLES
REAL STRENGTH
https://www.realwarriors.net/

The Real Warriors Campaign promotes a culture of support for psychological health by linking service members, veterans and their families with free, confidential resources including online articles, print materials, videos & podcasts.

Military Crisis Line
1-800-273-8255 PRESS 1
https://www.veteranscrisisline.net/get-help/military-crisis-line

The Military Crisis Line, text-messaging service, and online chat provide free VA support for all Service members, including members of the National Guard and Reserve, and all Veterans, even if they are not registered with VA or enrolled in VA health care.

SMARTPHONE APPLICATIONS

BREATHE2RELAX

Teaches breathing techniques as a tool for stress reduction. Available for iOS and Andriod.

Download on the App Store

GET IT ON Google Play

T2 MOOD TRACKER

Designed to help you track your emotional experience over time and provide you with a tool to share this information with your health care provider.

Download on the App Store

GET IT ON Google Play

BAMC/FSH RESOURCES

Department of Behavioral Health

Call for group start dates: (210)916-1600

Walk-In Services are available. Known emergencies should go to the nearest Emergency Room

Chaplain Family Life Center

Call for more information: (210)221-5007 OR (210)365-6420

Faith-based Counciling
Diverse Religious Services
Religious Education
Auxiliary Ministries

WRITE IT DOWN!
WHY DO YOU WANT TO MAKE YOUR EMOTIONAL HEALTH A PRIORITY?

CONTINUE ON BACK

Legal Disclaimer: The appearance of hyperlinks and downloadable applications does not constitute endorsement by the U.S. Army, of non-U.S. Government sites or the information products, or services contained therein. Although the U.S. Army may link to this site for informational purposes it does not exercise editorial control over the information that you may find on this site. Such links are provided consistent with the stated purpose of this document.

RESOURCES FOR ADDRESSING

EMOTIONAL & SPIRITUAL HEALTH

Empowering you to take control of your health and well-being

SMARTPHONE APPLICATIONS CONT.

VIRTUAL HOPE BOX

Contains tools to help patients with coping, relaxation, distraction, and positive thinking. Designed for use by patients and their BH providers as an accessory to treatment. The patient can then use the VHB away from clinic, continuing to add or change content as needed.

Download on the App Store

GET IT ON Google Play

LIFEARMOR

A comprehensive learning and self-management tool to assist members of the military community with common mental health concerns including sleep, depression, relationship issues, and post-traumatic stress.

Download on the App Store

GET IT ON Google Play

ADDITIONAL RESOURCES

BOOSTING RESILIENCE THROUGH SPIRITUALITY

BY: ARMY PUBLIC HEALTH CENTER

A brochure on how spirituality can help you feel connected to something bigger than yourself and build resilience. It covers topics such as:

- The benefits of spiritual fitness
- Spiritual fitness tips
- What leaders can do
- How to find a chaplain

WRITE IT DOWN!

WHAT ARE CONSIDERED EMOTIONAL AND OR SPIRITUAL HEALTH CHALLENGES?

"SPIRITUALITY CAN GIVES YOU A SENSE OF VALUE/PURPOSE AND STRENGTH/COMFORT DURING DIFFICULTY TIMES."

Legal Disclaimer: The appearance of hyperlinks and downloadable applications does not constitute endorsement by the U.S. Army, of non-U.S. Government sites or the information products, or services contained therein. Although the U.S. Army may link to this site for informational purposes it does not exercise editorial control over the information that you may find on this site. Such links are provided consistent with the stated purpose of this document.

Mobile Apps created and/or endorsed by US government agencies

General Use

Name	Description	Android QR	iOS QR
Performance Triad<br>From TRADOC Mobile	Resources and information on Sleep, Activity, and Nutrition based on roles: Soldier, Civilians, Pre-Retiree/Retiree. Ability to monitor Sleep, Activity, Nutrition Targets		
Digital Garrison<br>From Army & Airforce Exchange Services	Explore all the benefits your local Army post has to offer; events, shops, clinics, activity centers, and directorates. Navigate to on-post facilities, stay updated on local weather, and be informed about gate closures or other important installation information in real time.		
P3<br>From Army Public Health	The Performance Triad contains practical information and resources on how to fit healthy sleep, activity, and nutrition into your everyday life. Using the Performance Triad's scientifically proven principles of sleep, activity, and nutrition to target changes in your daily routine will help you become stronger, faster, leaner, and mentally sharper.		

Sleep

Name	Description	Android QR	iOS QR
CBT-I Coach<br>From US Department of Veteran Affairs<br>***	Helps guide users through the process of learning about sleep, developing positive sleep routines, and improving their sleep environments. CBT-i Coach is intended to augment face-to-face care with a healthcare professional. It can be used on its own, but it is not intended to replace therapy for those who need it.		
CPT Coach<br>From US Department of Veteran Affairs<br>***	For Veterans, Service members, and others with PTSD who are participating in Cognitive Processing Therapy (CPT) with a professional mental healthcare provider. The app contains support materials for a complete course of CPT to help patients manage their treatment, including between session assignments, readings, PTSD symptom monitoring, and mobile versions of CPT worksheets. Not intended to replace therapy for those who need it		
Insomnia Coach<br>From US Department of Veteran Affairs	For Veterans, military Servicemembers, and others who are suffering from insomnia. It is designed to be used daily for 5 weeks with a sleep coach by following the Training Plan and sleep diary. This app is based on scientific research about how people can change their behaviors and thoughts to improve their sleep. This app does NOT replace professional care.		

Breath2Relax<br>From T2	Breathe2Relax is a hands-on diaphragmatic breathing exercise. Breathing exercises have been documented to decrease the body's 'fight-or-flight' (stress) response, and help with mood stabilization, anger control, anxiety, and sleep management		

Activity

Name	Description	Android QR	iOS QR
MissionFit<br>From Defense Health Agency	This app provides a 12 week program of exercise routines, along with library of 90+ exercises with detailed instructions including video, images and textual description. Users are guided through the workout routines by navigating workout weeks, days and exercise routine instructions.		
Army PRT<br>From TRADOC Mobile	This app prescribes the method for the execution of the Army Physical Readiness Training System. Features include sample training schedules with drill and exercise demos, body composition calculators, an APRT calculator, a metronome, a run timer		
Official Navy PFA<br>From SeaWarrior Applications	Provides Sailors with all the Physical Readiness Program information they need to maintain optimal health, fitness, and readiness according to Navy standards. The app offers current guidance regarding all aspects of the Navy's Physical Readiness Program, including information on appropriate nutrition, health, fitness, aerobic capacity, muscular strength, muscular endurance, and body fat composition.		
Fitness Preparation<br>From SAIC - Science Applications International Corp	This app is focused on the physical fitness portion of this ideology: improving physical performance through training and nutrition. It provides photographs and descriptions of exercises used by MARSOC, and will help prepare candidates for the physical aspects of the Assessment and Selection process. Upon arriving at A&S, candidates are expected to have completed this 10-week program		
MOVE! Coach<br>From US Department of Veterans Affairs (VA)	A weight management app designed for Veterans, service members, their families, and others who strive for a healthy weight. The 19-week program guides users to achieve success through education and the use of tools, in an easy and convenient way. Participants can monitor, track, and receive feedback regarding their progress with weight, diet, and exercise goals.		

Army Health & Fitness<br>From Army Public Health	Army Health and Fitness is a digital quarterly publication that highlights trending health topics that impact Soldiers and their Family. It is an interactive experience that features articles, videos, animations, and quizzes. The Army Health and Fitness team is committed to delivering authentic information that informs and inspires.		
NIH BMI Calculator	The National Heart, Lung, and Blood Institute's BMI (Body Mass Index) calculator is a useful tool to screen for weight categories that may lead to health problems. The downloadable phone application puts the fully functioning calculator right on your phone, along with links to resources on the NHLBI site.		

Nutrition

Name	Description	Android QR	iOS QR
Start Simple with MyPlate<br>From USDA Food & Nutrition Service	Quickly and easily track not only calories but also quantities of each food group so you can be sure you are getting a balanced diet on the go. See a list of what you eat each day with quick-glance charts that compare actual consumption to your custom MyPlate food plan		
Fooducate - Eat better. Lose weight.<br>From Fooducate, Ltd.	Automatically grades foods and beverage on a scale from A to D. Fooducate's algorithm is based on information that is publicly available on a product's package: the nutrition facts panel and the ingredient list. We do not receive any additional information from manufacturers.		
Amerifit Nutrition Tracker<br>From Fooducate, Ltd.	The Amerifit nutrition tracker app powered by Fooducate helps you lose weight, track your progress, and eat REAL food. Amerifit functions as well at home as it does at work or on campus. Scan a product barcode anywhere or QR code in your café to see what's really in your food. Amerifit will also show you healthier alternatives!		
Calorie Counter – MyFitnessPal<br>MyFitnessPal, Inc.	Log your calories, monitor water intake, monitor carbohydrate and proteins. Remembers what you've eaten and done most often in the past, and makes it easy for you to add those foods again to your log.		

Additional

Name	Description	Android QR	iOS QR
Covid Coach<br>US Department of Veterans Affairs (VA)	COVID Coach is designed to help you build resilience, manage stress, and increase your well-being during this crisis. The app is free, secure, and helps connect you to important resources for coping and adapting during the COVID-19 pandemic		
Battle Buddy.<br>From TRADOC Mobile	This app helps you be good Battle Buddy by giving you the tools and information needed to assist your buddy during a crisis. It also provides detailed intervention, awareness and prevention information concerning the Army's suicide prevention program and Sexual Harassment/Assault Response and Prevention program.		
Provider Resilience<br>From T2	Provider Resilience gives frontline providers tools to keep themselves productive and emotionally healthy as they help our nation's service members, veterans, and their families.		
Stay Quit Coach<br>US Department of Veterans Affairs (VA)	This app help track your cravings and moods, monitor your progress toward achieving smoke free milestones, identify your smoking triggers, upload personalized "pick me ups" to use during challenging times to help you successfully become and stay smoke free		
LifeArmor<br>From T2	LifeArmor is a comprehensive learning and self-management tool to assist members of the military community with common mental health concerns. Built for both iOS and Android, LifeArmor is portable and provides information and assistance at the touch of a button.		
QuitGuide<br>ICF International	Track your cigarette cravings and moods, monitor your progress toward achieving smoke free milestones. Discover your reasons for quitting smoking, get expert guidance on how to quit smoking and address nicotine withdrawal, and access a variety of other strategies to help you successfully become and stay smokefree.		
Moving Forward<br>US Department of Veterans Affairs (VA)	Provides tools and teaches problem solving skills to overcome obstacles and deal with stress. The app is designed for Veterans and Service members, but is useful for anyone with stressful problems. It is especially helpful in managing challenges such as: returning to civilian life, balancing school and family life, financial difficulties, relationship problems, difficult career decisions, and coping with physical injuries. It may be used alone or in combination with the Moving Forward online course	Not Available	

Couple's Coach<br>US Department of Veterans Affairs (VA)	Designed for partners who want to improve their relationship and explore new ways to connect. It takes users through five levels of expert-written education and engaging behavioral exercises informed by science. Learn about different approaches to common relationship issues and review available resources in their communities. It also includes comprehensive relationship information for couples living with PTSD. It isn't a replacement for face-to-face couples counseling.		
Sesame Street for Military Families<br>From Sesame Workshop	Developed for parents and caregivers: Use your mobile device to access engaging videos, articles, storybooks, parent guides, and more to help you support your preschool and school-aged children as they encounter transitions common to military families.		
The Big Move<br>From Sesame Workshop	This is an app for families coping with a recent or pending move, which will help teach your child (ages 2–5) about what to expect when relocating. The Parents Section contains more detailed tips and suggestions on; hearing the news, packing, saying goodbye, expressing feelings, traveling, exploring the new home, and making new friends.		
Parenting2Go<br>US Department of Veterans Affairs (VA)	Parenting2Go helps Veterans and Service members reconnect with their children and provides convenient tools to strengthen parenting skills. Parents can find quick parenting advice; relaxation tools to use when frustrated or stressed; tools to improve their relationship with their children through positive communication; and strategies to switch gears between military life and home.	Not Available	
Mindfulness Coach<br>US Department of Veterans Affairs (VA)	This app was developed to help Veterans, Service members, and others learn how to practice mindfulness. It offers exercises, information, and a tracking log to optimize your practice. Mindfulness has been shown to be effective for reducing stress, improving emotional balance, increasing self-awareness, helping with anxiety and depression, and coping more effectively with chronic pain.		
T2 Mood Tracker<br>From T2	T2 Mood Tracker is a mobile application that allows users to monitor and track emotional health. Originally developed as a tool for service members to easily record and review their behavior changes, particularly after combat deployments, it has now become very popular with many civilian users around the world.		

Tactical Breather<br>From T2	The Tactical Breather application can be used to gain control over physiological and psychological responses to stress. Through repetitive practice and training, anyone can learn to gain control of your heart rate, emotions, concentration, and other physiological and psychological responses to your body during stressful situations.		
Virtual Hope Box<br>From T2	Designed for use by patients and their behavioral health providers as an accessory to treatment. The VHB contains simple tools to help patients with coping, relaxation, distraction, and positive thinking. Patients and providers can work together to personalize the VHB content on the patient's own smartphone according to the patient's specific needs.		
PTSD Coach<br>US Department of Veterans Affairs (VA)	This app provides users with education about PTSD, information about professional care, a self-assessment for PTSD, opportunities to find support, and tools that can help users manage the stresses of daily life with PTSD. Tools range from relaxation skills and positive self-talk to anger management and other common self-help strategies. Users can customize tools based on their preferences and can integrate their own contacts, photos, and music. This app can be used by people who are in treatment as well as those who are not.		

Recommended Websites

Name	Description	URL
**General**		
**Military One Source**	24/7 connection to information, answers and support to help you reach your goals, overcome challenges and thrive. Turn here for tax services, spouse employment help, webinars and online training, relocation and deployment tools, and much more.	https://www.militaryonesource.mil/
**HPRC**	Your source for military-specific evidence-based Total Force Fitness information. Find countless articles and resources on physical training, performance, resilience, sleep, stress management, mental health, nutrition, and family life.	https://www.hprc-online.org/
**Performance Triad (P3)**	Learn how to change your daily sleep, activity, and nutrition routines. Become stronger, faster, leaner, and mentally sharper.	https://p3.amedd.army.mil/
**Army MWR**	Family and MWR, seeks to bridge the gap between the garrison and the local community, and contribute to the Army's strength and readiness by offering services that reduce stress, build skills and self-confidence for Soldiers and their families.	https://www.armymwr.com/
**Tricare**	Find information of what is covered under your healthcare plan.	https://www.tricare.mil/
**Sleep**		
**Sleep By The National Sleep Foundation**	Find information about sleep through graphics and articles on how to help yourself get the right amount of sleep	https://www.sleep.org/
**Sleep Education by AASM**	Find articles, information, and helpful tools for better sleep. Includes links to national sleep campaigns and blogs that cover many sleep topics.	http://sleepeducation.org/
**NIH Sleep Guide**	Your free guide to healthy sleep from the NIH	https://www.nhlbi.nih.gov/files/docs/public/sleep/healthy_sleep.pdf
**Veterans Affairs Path to Better Sleep**	Path to Better sleep is the latest, free online training course. Created by VA sleep experts, Path to Better Sleep was designed with Veterans in mind to identify and treat Insomnia and screen for other sleep disorders.	https://www.veterantraining.va.gov/Insomnia/
**Activity**		
**Everybody WALK**	Find information about the health benefits of walking, news, videos, & local events	https://everybodywalk.org/
**NIH Walking Program**	A easy to follow sample of an 8 week walking program	https://www.nhlbi.nih.gov/health/educational/healthdisp/pdf/tipsheets/Sample-Walking-Program.pdf
**Alltrails**	App and website with info about thousands of walking, hiking and biking trails all over the world.	https://www.alltrails.com/
**MOVE Your Way! By Health.gov**	Find tools, videos, and fact sheets to help you make small changes in your activity level to reap big health benefits!	https://health.gov/moveyourway
**Nutrition**		
**Fooducate**	A companion website to the FOODUCATE app that gives resources on	https://www.fooducate.com/
**P3's Cook Book**	A cook book created by the Performance Triad Team	https://cookpad.com/us/users/2940124
**Choose My Plate From USDA Food & Nutrition Service**	MyPlate helps find your healthy eating style and build it throughout your life. Everything you eat and drink matters. The right mix can help you be healthier now and in the future.	https://www.choosemyplate.gov/

Intrinsic		
**Moving Forward US Department of Veterans Affairs (VA)**	Helps you face common challenges such as: stress management, relationship problems, balancing school and work, financial difficulties and adjustment issues.	https://www.veterantraining.va.gov/movingforward/
**Borne the Battle Podcast**	Available on itunes, google, and sticher	https://www.blogs.va.gov/VAntage/borne-the-battle-podcast/
**Blue GRIT Podcast**	Features conversations with current and former military leaders, mental health experts, elite athletes, veterans and other individuals who have overcome significant adversity. Podcast increases awareness of psychological health and assists in de-stigmatizing mental illness, normalizing struggles and internalizing the message of hope and recovery.	https://soundcloud.com/user-52299767
**You can QUIT 2**	An educational campaign for the U.S. military. The mission of the campaign is to help U.S. Service members quit tobacco—for themselves and their loved ones. The site supports the DOD's efforts to build and sustain a ready and resilient force by providing resources for Service members, as well as their family and friends and health professionals.	https://www.ycq2.org/
**DOD Safe Helpline**	Safe Helpline is the Department of Defense's (DoD) sole hotline for members of the DoD community affected by sexual assault. Safe Helpline is a completely anonymous, confidential, 24/7, specialized service—providing help and information anytime, anywhere.	https://www.safehelpline.org/
**Real Warriors Campaign**	Promotes a culture of support for psychological health by encouraging the military community to reach out for help whether coping with the daily stresses of military life, or concerns like depression, anxiety and posttraumatic stress disorder.	https://www.realwarriors.net/
**Military Crisis Line**	A free, confidential resource that's available to anyone, even if you're not registered with VA or enrolled in VA health care. The caring, qualified responders at the Veterans Crisis Line are specially trained and experienced in helping Veterans of all circumstances.	https://www.veteranscrisisline.net/
**Extrinsic**		
**Happify Daily**	Free portion of Happify with blog posts relating to health, personal development, mindset, behavior, coping, resilience, etc.	https://happify.com/public/happify-daily/
**ARMY SHARP**	The Army's campaign to combat sexual harassment and sexual assault by engaging all Soldiers in preventing sexual assault before they occur.	https://www.preventsexualassault.army.mil/
**Army Community Resources**	Online directory to your local Army installation. List of programs and services available to members of US military communities including military service members, their family members, DoD civilian personnel and retirees. Expand individual resources for additional information including websites, email addresses, and phone numbers.	https://crg.amedd.army.mil/Pages/default.aspx
**Military Kids Connect**	Online community for military children ages 6-17. Provides access to age-appropriate resources to support children dealing with the unique psychological challenges of military life. MKC offers informative activities, helpful videos, and a community that can build and reinforce understanding, resilience, and coping skills in military children and their peers.	https://militarykidsconnect.health.mil/
**Sesame for Military Families**	This critical outreach tool helped military families and their young children cope with the challenges of deployment and build resilience in times of separation and change.	https://sesamestreetformilitaryfamilies.org/
**Parenting2GO by Veterans Affairs**	Built by the Department of Veterans Affairs (VA) providing a free online site for military and Veteran parents with information and strategies to improve their parenting skills.	https://www.veterantraining.va.gov/apps/veteranparenting/index.html
